# Supplementary material for: TM9SF4 is an F-actin disassembly factor that promotes tumor progression and metastasis
Source: Nat Commun. 2022 Sep 29;13:5728. doi: 10.1038/s41467-022-33276-y (PMC9522921; doi:10.1038/s41467-022-33276-y)
Supplement: Supplementary file 1 — Supplementary Information [file 41467_2022_33276_MOESM1_ESM.pdf]

Supplementary information for:

TM9SF4 is an F-actin disassembly factor that promotes tumor progression and metastasis

Zhaoyue Meng[1,2,4], Zhichao Li[1,3,4], Mingxu Xie[1,4], Hongyan Yu[1], Liwen Jiang[2]*, Xiaoqiang Yao[1,2]*

[1] School of Biomedical Sciences and Li Ka Shing Institute of Health Sciences, Faculty of Medicine, The Chinese University of Hong Kong, Hong Kong, China

[2] Centre for Cell & Developmental Biology and State Key Laboratory of Agrobiotechnology, School of Life Sciences, The Chinese University of Hong Kong, Hong Kong, China

[3] Key Laboratory of Medical Reprogramming Technology, Shenzhen Second People's Hospital, The First Affiliated Hospital of Shenzhen University, Shenzhen, China

[4] These authors contributed equally

* Correspondence: yao2068@cuhk.edu.hk, ljiang@cuhk.edu.hk

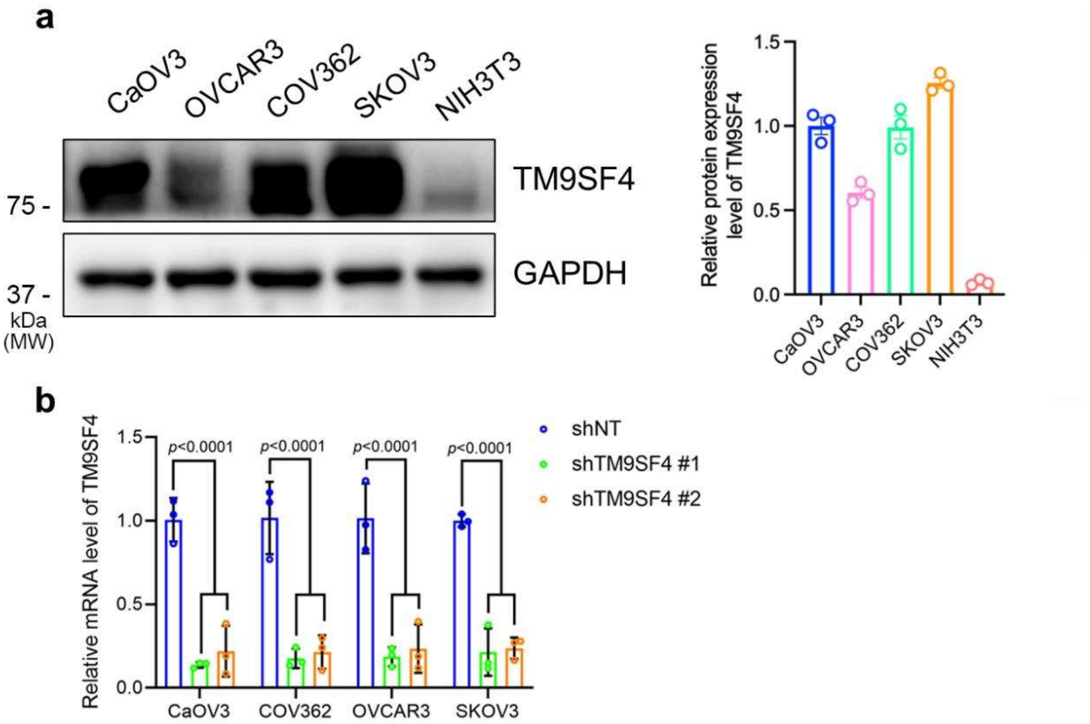

Fig. S1 | TM9SF4 is highly expressed in HGSOC cell lines. a Shown are representative immunoblot images (left) and summary data (right) of endogenous expression level of TM9SF4 in different HGSOC cell lines. **b** Knockdown efficiency of TM9SF4-shRNAs in different HGSOC cell lines was measured by RT-qPCR. Data are presented as mean ± SEM from 3 biologically independent experiments and two-tailed unpaired Student's t-test was used for statistical analysis. Source data are provided as a Source Data file.

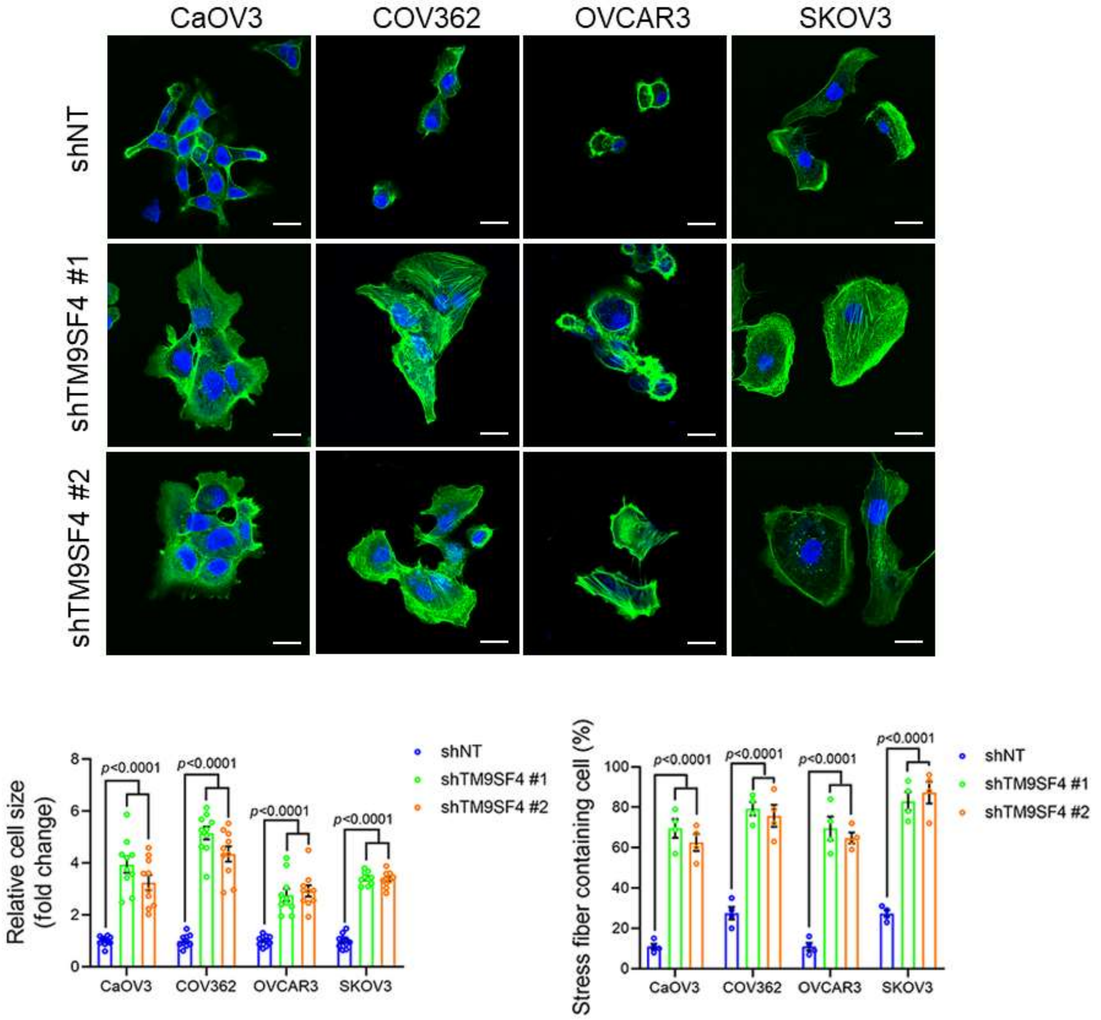

Fig. S2 | Knockdown of TM9SF4 increases the cell size and promotes F-actin formation in multiple ovarian cancer cell lines. TM9SF4 knockdown by lenti-shRNAs increased the cell size and F-actin stress fiber formation in CaOV3 cells, COV362 cells, OVCAR3 cells and SKOV3 cells. Shown are representative confocal microscope images after FITC-phalloidin staining (upper) and summary data (lower). DNA was visualized by DAPI staining. Scale bar = 10 μm. Data are shown as mean ± SEM (n = 3 biologically independent experiments, >200 cells from 3-5 different fields per experiment) and two-tailed unpaired Student's t-test was used for statistical analysis. Source data are provided as a Source Data file.

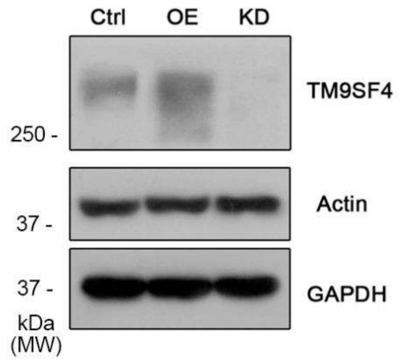

Fig. S3 | Overexpression or knockdown of TM9SF4 have no effect on total actin levels. Expression of TM9SF4, actin, and GAPDH in control or TM9SF4 overexpression (OE/KD A2780 cells by immunoblots. Shown were representative images. The experiments were repeated three times with similar results. OE, overexpression; KD, knockdown. Source data are provided as a Source Data file.

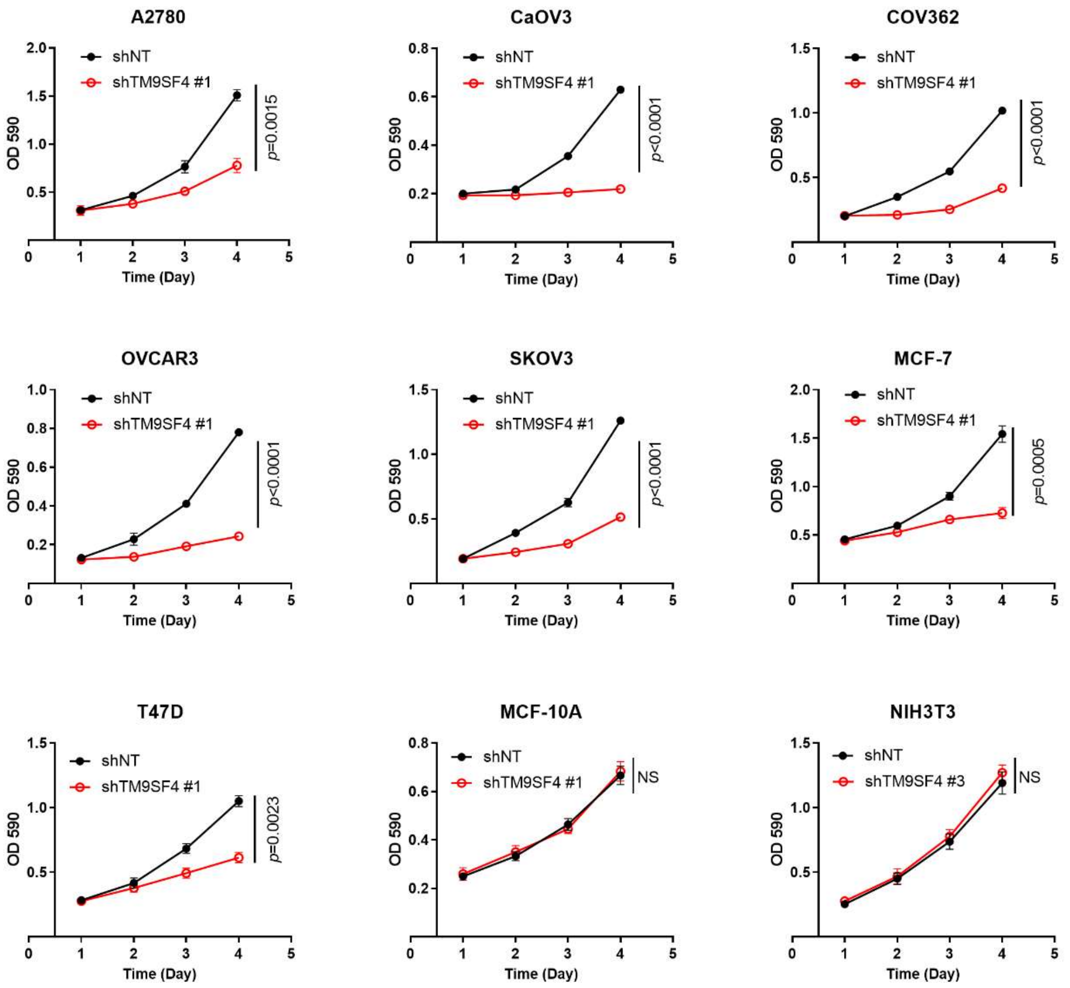

Fig. S4 | Knockdown of TM9SF4 reduces cell population growth of cancer cell lines. The cells were treated with lentivirus-based shTM9SF4 #1 or shNT (control), followed by measurement of cell population growth using MTT assay. Data are shown as mean ± SEM from 3 biologically independent experiments and two-tailed unpaired Student's t-test was used for statistical analysis. NS, no significant difference. Source data are provided as a Source Data file.

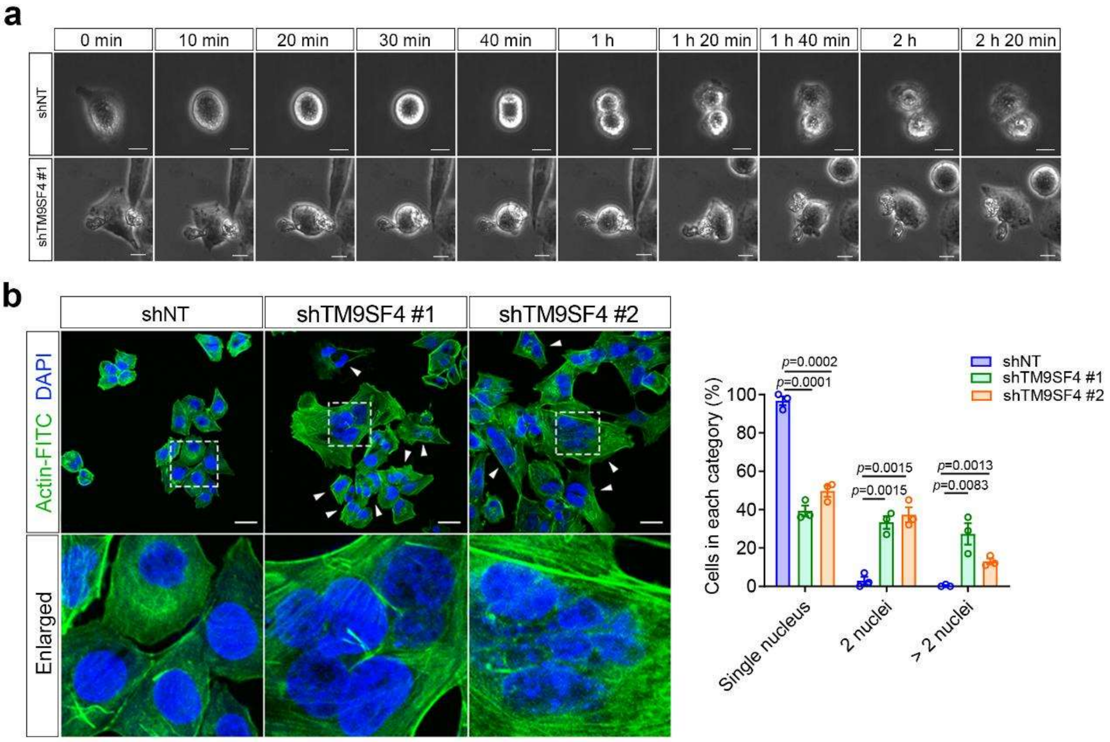

Fig. S5 | Knockdown of TM9SF4 causes defective cytokinesis. a Time-lapse analysis of cytokinesis of control and TM9SF4 knockdown A2780 cells. Frames "0 min" represent metaphase. Control cells underwent cell division and spreading after the metaphase, whereas the process in TM9SF4 knockdown cells were largely impaired. Scale bar = 5 μm. **b** Control and TM9SF4 knockdown A2780 cells were fixed, and DNA was visualized by DAPI staining. Representative images (left) of TM9SF4 knockdown cells and summary data (right) showing the formation of multinucleated cells. Scale bar = 10 μm. Data are presented as mean ± SEM from 3 biologically independent experiments and two-tailed unpaired Student's t-test was used for statistical analysis. Source data are provided as a Source Data file.

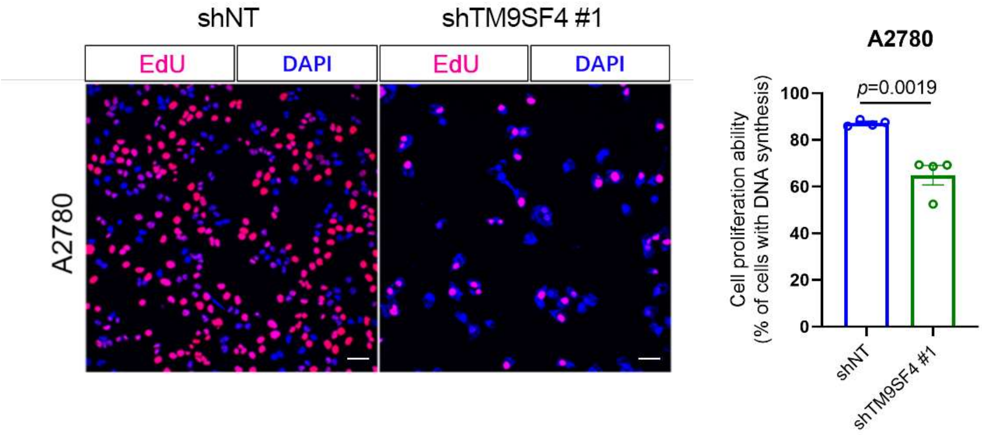

Fig. S6 | Cell proliferation assay (EdU) after shRNA knockdown in A2780 cells. Cell proliferation assay (EdU) of A2780 cells, treated with lentivirus-based scrambled shRNA or TM9SF4 shRNA. EdU is Red, while DAPI is in Blue. Scale bar = 10 µm. Data are presented as mean ± SEM from 4 biologically independent experiments and two-tailed unpaired Student's t-test was used for statistical analysis (>300 cells from 3 different fields per experiment). Source data are provided as a Source Data file.

a

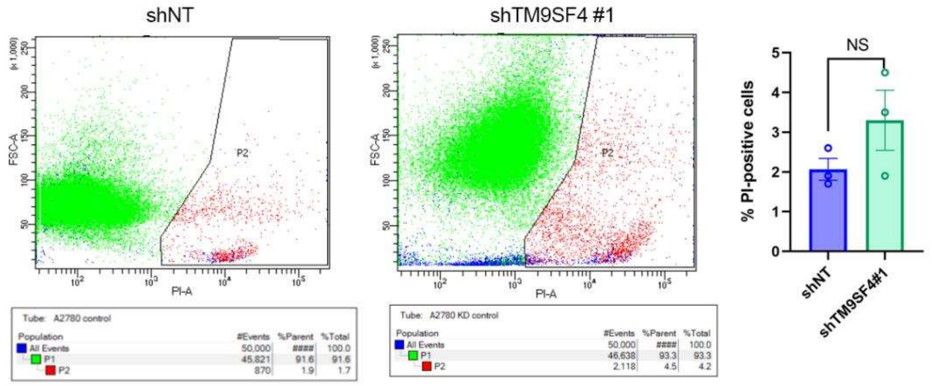

b

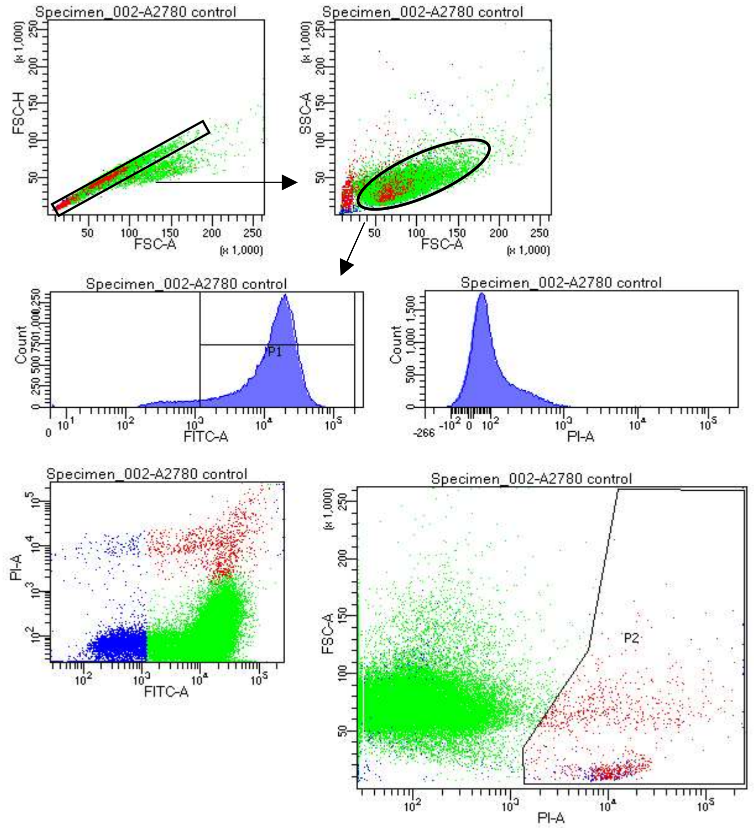

Fig. S7 | Cell death assay after shRNA knockdown in A2780 cells. a Representative images of cell death determined by a flow cytometry-based PI staining. A2780 cells were treated with lentivirus-based scrambled shRNA (left) or TM9SF4 shRNA (middle), collected and fixed for detection. Right panel is summary. P2 fraction represented PI-positive dead cells. Data are presented as mean ± SEM from 3 biologically independent experiments and two-tailed unpaired Student's t-test was used for statistical analysis. NS, not significant. **b** Representative flow cytometry plots show gating strategy to identify PI-positive cell subsets. Source data are provided as a Source Data file.

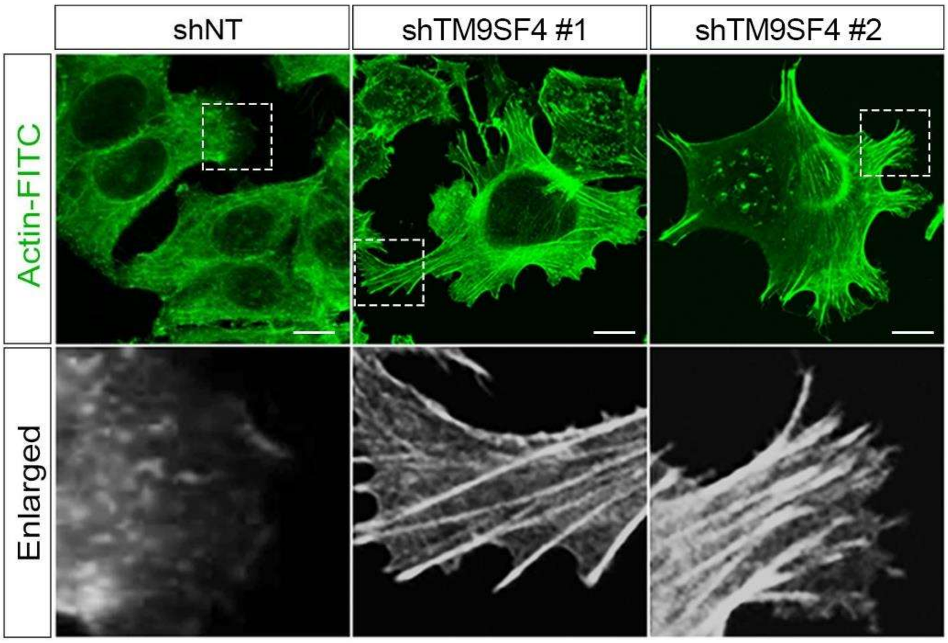

Fig. S8 | Knockdown of TM9SF4 induces actin stress fiber formation. MCF-7 cells were treated with lentivirus-based shTM9SF4 #1 or shTM9SF4 #2, or control shNT. F-actin was visualized by FITC-phalloidin staining. Shown are representative images of cells from each population. The boxed regions are enlarged, showing the stress fiber formation at cell periphery. Scale bar = 5 μm. The experiments were repeated three times with similar results.

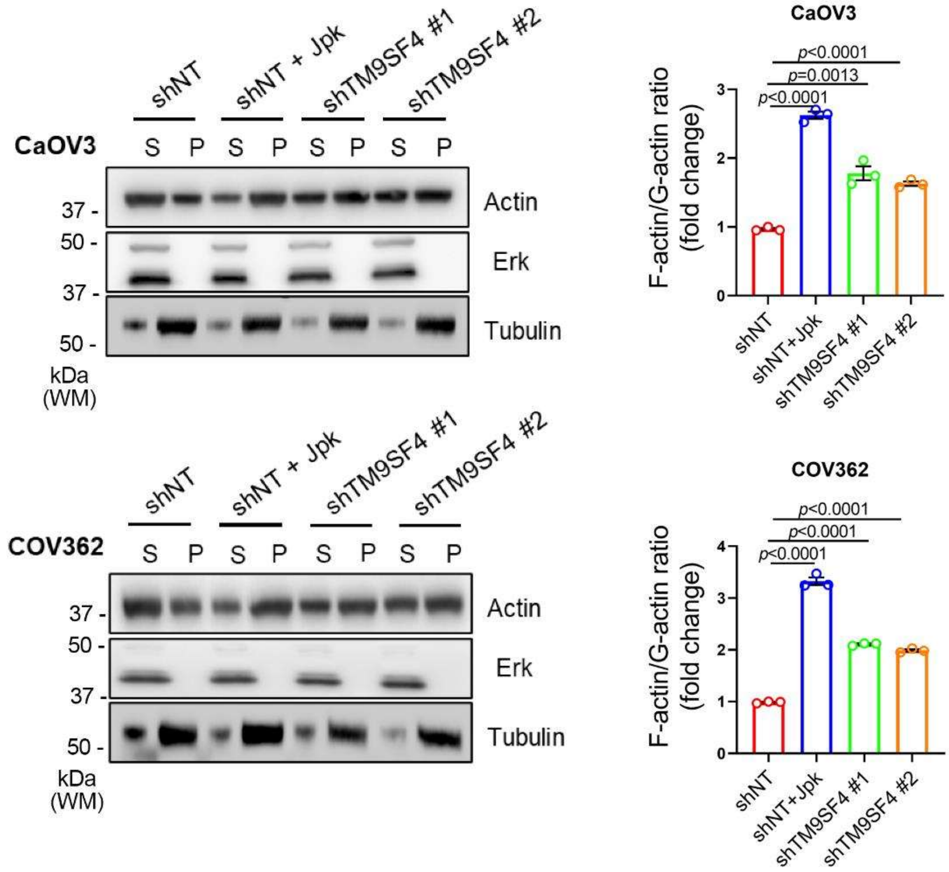

Fig. S9 | TM9SF4 knockdown increases the proportion of F-actin in CaOV3 cells and COV362 cells. Shown are representative immunoblot images and summary data of soluble actin (G-actin) in the supernatant fraction (S) and insoluble actin (F-actin) in the pellet fraction (P). ERK, cytosolic marker; β-tubulin, cytoskeleton marker. Data are presented as mean ± SEM from 3 biologically independent experiments. Two-tailed unpaired Student's t-test was used for statistical analysis. Source data are provided as a Source Data file.

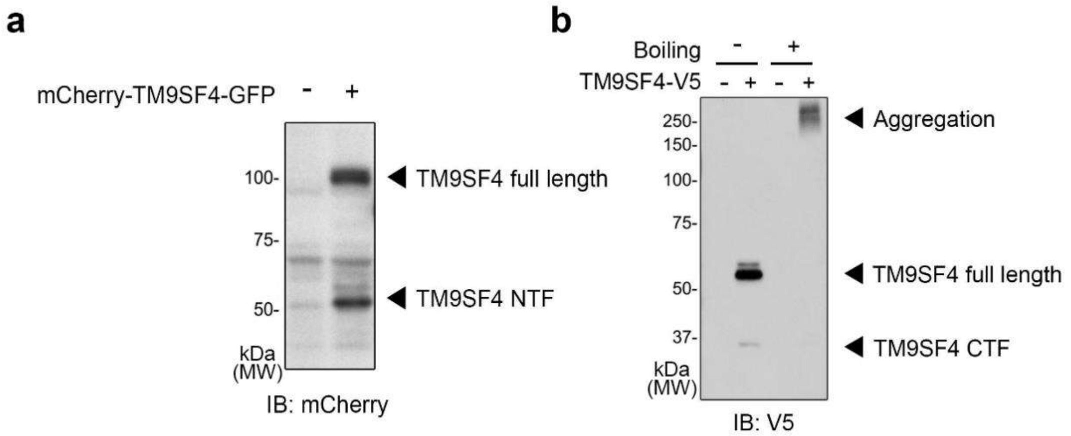

Fig. S10 | Identification of an N-Terminal fragment of TM9SF4 in A2780 cells. a Representative western blot analysis showing an N-terminal fragment detected by anti-mCherry antibody in mCherry-TM9SF4-GFP expressing A2780 cells. **b** Immunoblot analysis of whole cell lysates from TM9SF4-V5 expressing A2780 cells. A V5-tagged TM9SF4 CTF was detected under mild denature condition. Shown were representative images. The experiments were repeated three times with similar results. Source data are provided as a Source Data file.

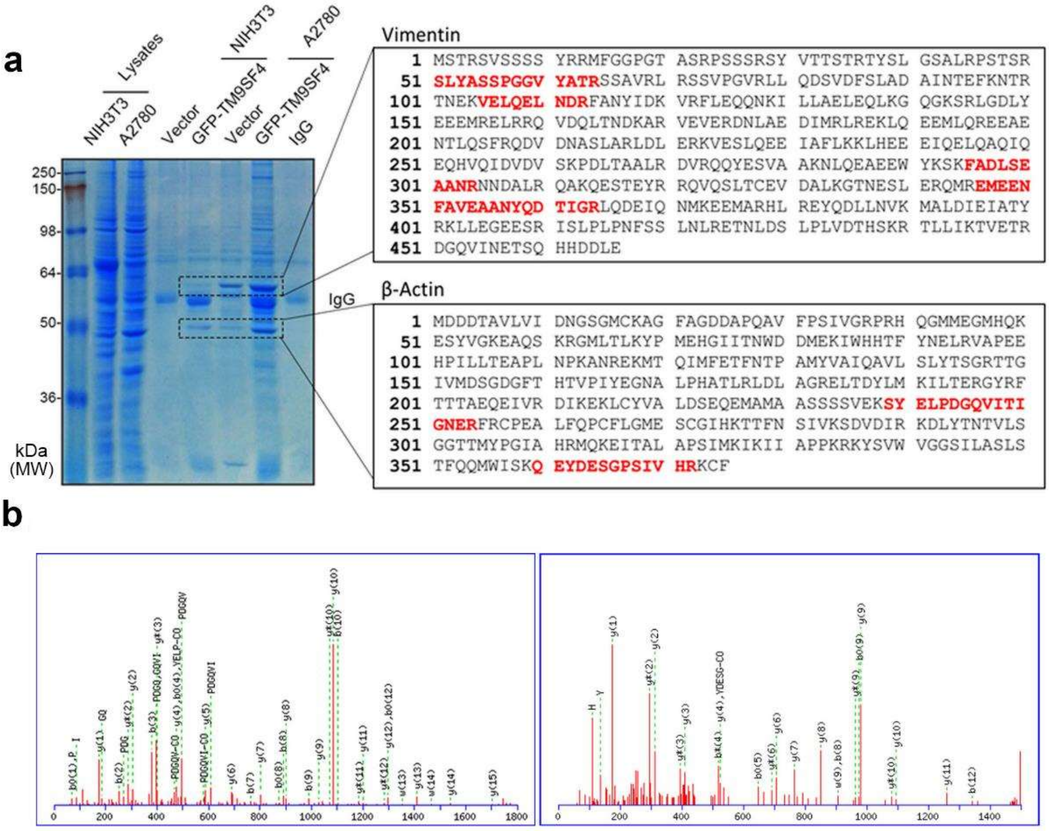

Fig. S11 | Identification of β-actin as an interaction partner of TM9SF4. a Mass spectrometry results of the anti-TM9SF4 or anti-GFP immunoprecipitations in NIH3T3 and A2780 cells. The protein segments highlighted in red were detected by MS/MS. **b** Representative MS/MS fragmentation of β-actin peptide. The experiments were repeated three times with similar results. See also Supplementary Data 1.

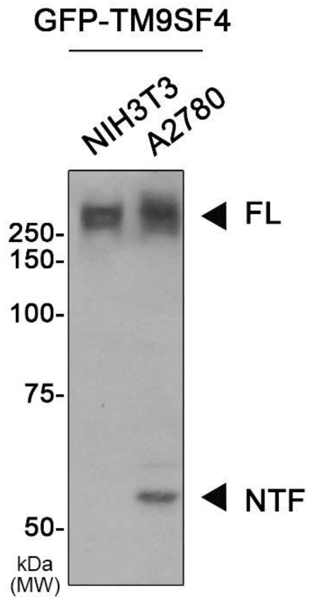

Fig. S12 | Full-length TM9SF4 can be cut to release an NTF in A2780 cells but not in NIH3T3 cells. Western blots showing a truncated NTF can only be detected in A2780 cells but not in NIH3T3 cells. The experiments were repeated three times with similar results. Source data are provided as a Source Data file.

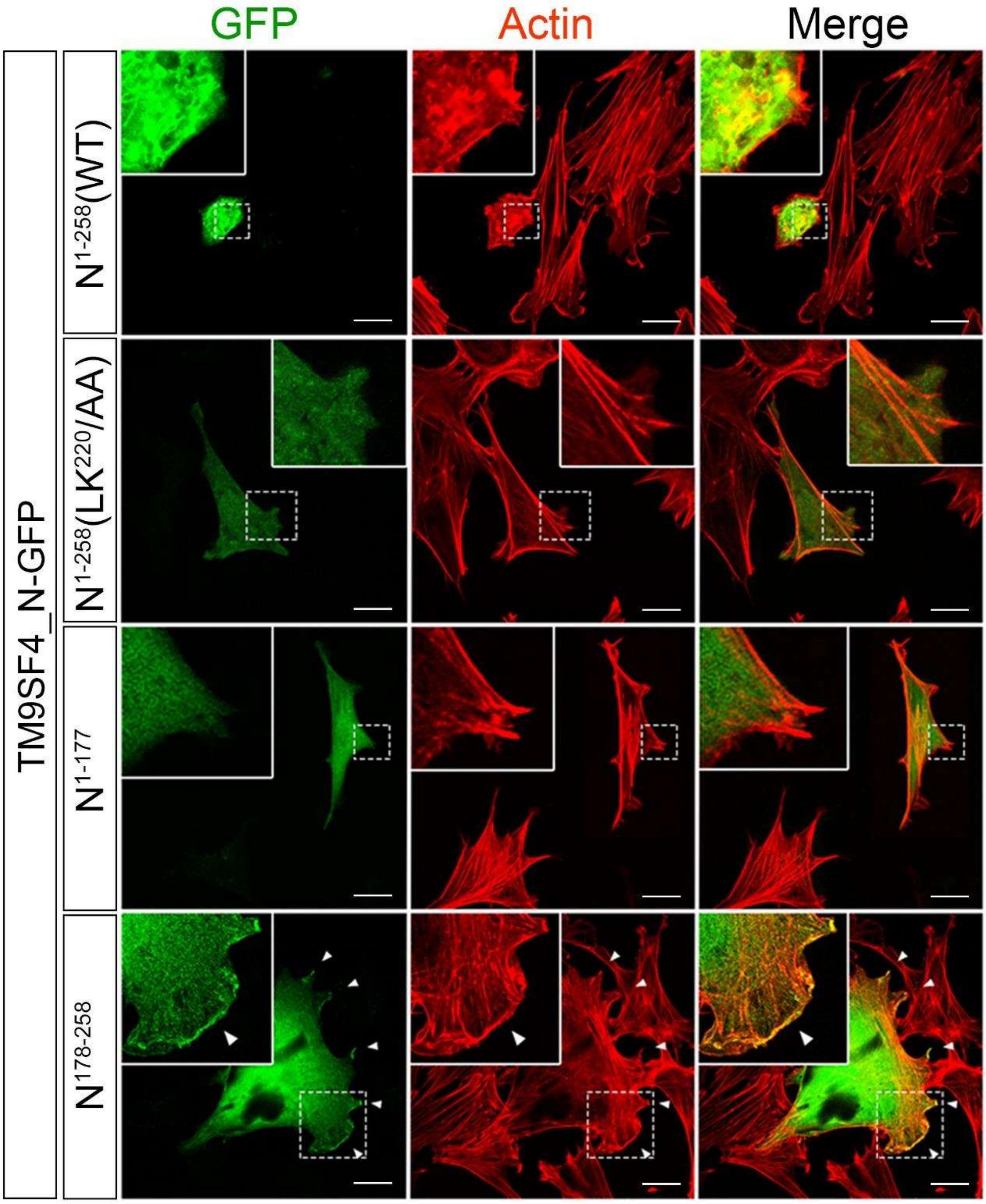

Fig. S13 | The ABD domain of TM9SF4 NTF is responsible for actin binding. TM9SF4_N$^{1\text{-}258}$-GFP, TM9SF4_N$^{1\text{-}258}$ (LK220/AA)-GFP, TM9SF4_N$^{1\text{-}177}$-GFP, and TM9SF4_N$^{178\text{-}258}$-GFP were transiently expressed in NIH3T3 cells. The cells were fixed and stained with rhodamine phalloidin to visualize F-actin cytoskeleton. The colocalization of TM9SF4 NTF and F-actin was indicated by the white arrow heads. Scale bar = 10 μm. The experiments were repeated three times with similar results.

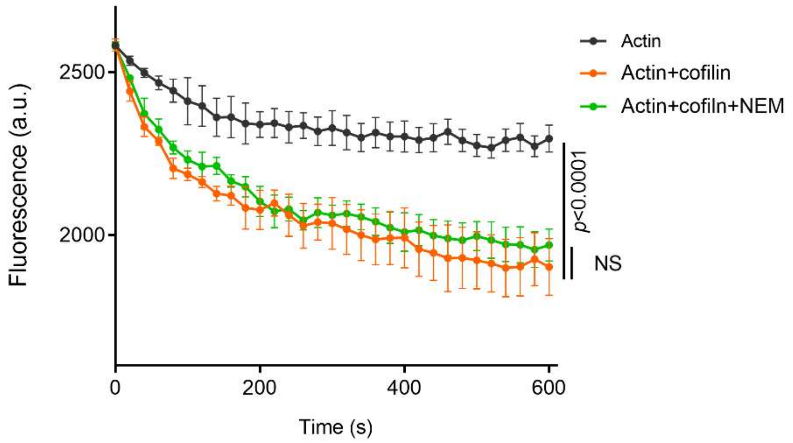

Fig. S14 | Analyses of NEM's effects on cofilin-mediated actin disassembly. Pyrene-actin depolymerization assay showing that NEM (2 mM) has no obvious effect on cofilin-mediated actin disassembly. F-actin (2.5 μM) were incubated with cofilin (1 μM) with or without NEM (2 mM), followed by measurement of fluorescent intensity change of pyrene-labeled F-actin. F-actin alone served as a control. Data are presented as mean ± SEM from 3 biologically independent experiments. Source data are provided as a Source Data file.

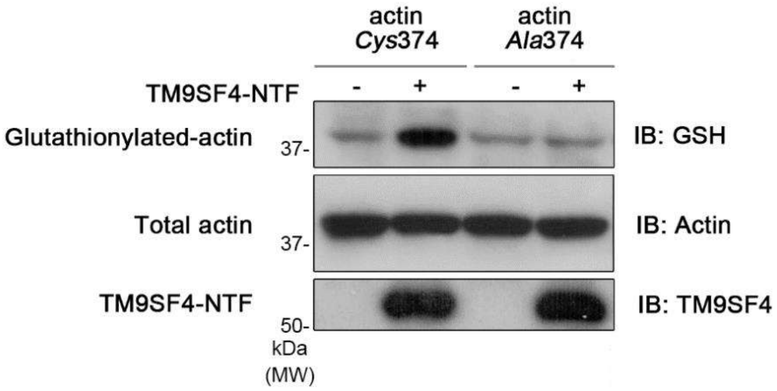

Fig. S15 | Actin Cys374 is essential for TM9SF4-NTF-induced actin oxidation/glutathionylation. F-actin glutathionylation after incubation of F-actin (2.5 μM, F-actin WT or F-actin C374A) with GST-TM9SF4_N$^{24-258}$ (1 μM) in the presence of 10 mM GSH. The samples were resolved in SDS-PAGE and immunoblotted with anti-GSH antibody. The experiments were repeated three times with similar results. Source data are provided as a Source Data file.

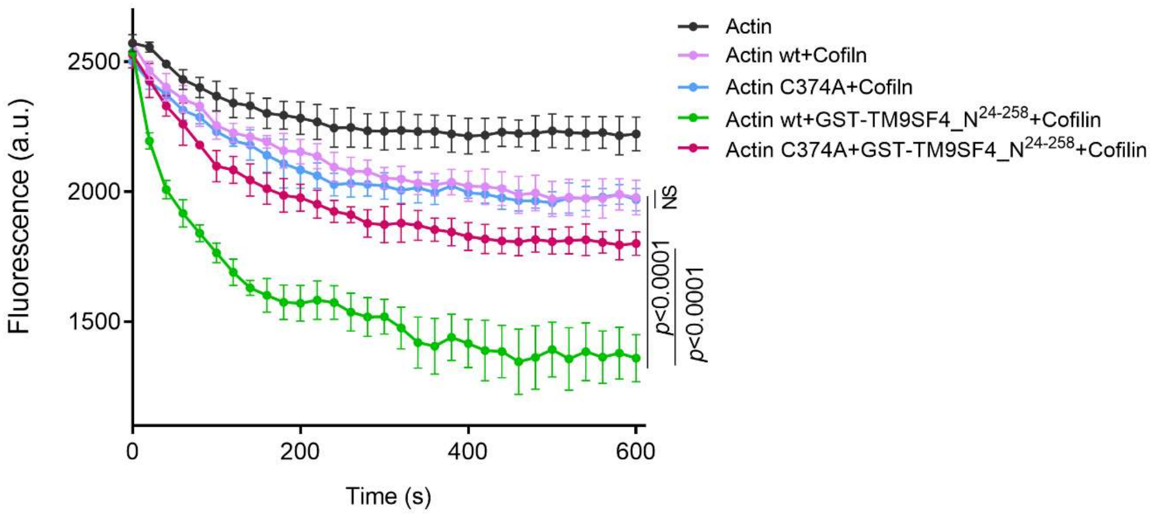

Fig. S16 | Actin Cys374 is involved in cofilin-mediated actin disassembly. Pyrene-actin depolymerization assay showing that GST-TM9SF4_N$^{24\text{-}258}$ induced cofilin-mediated actin disassembly, which was prevented by point mutation of actin Cys374 to Ala374. F-actin (2.5 μM, F-actin WT or F-actin C374A) were incubated with cofilin (1 μM) with or without GST-TM9SF4_N$^{24\text{-}258}$ (2 μM), followed by measurement of fluorescent intensity change of pyrene-labeled F-actin. Data are presented as mean ± SEM from 3 biologically independent experiments. Source data are provided as a Source Data file.

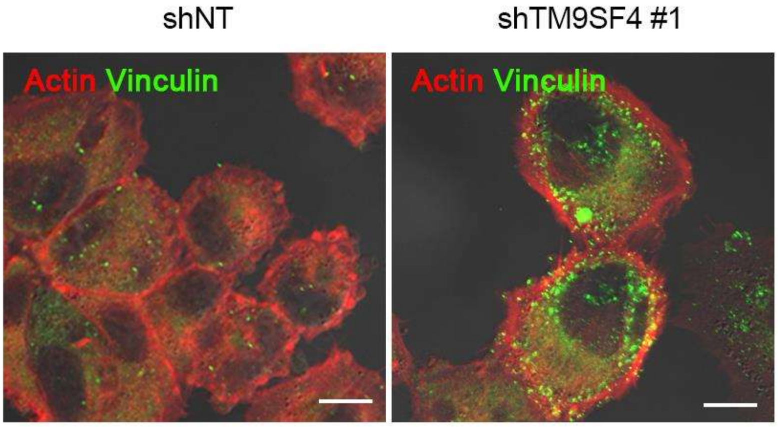

Fig. S17 | TM9SF4 suppression enhances A2780 cell adhesion. Representative images showing vinculin-associated focal adhesions in control cells (left) or cells with TM9SF4 shRNA (right), as detected by anti-vinculin antibody. Scale bar = 10 μm. The experiments were repeated three times with similar results.

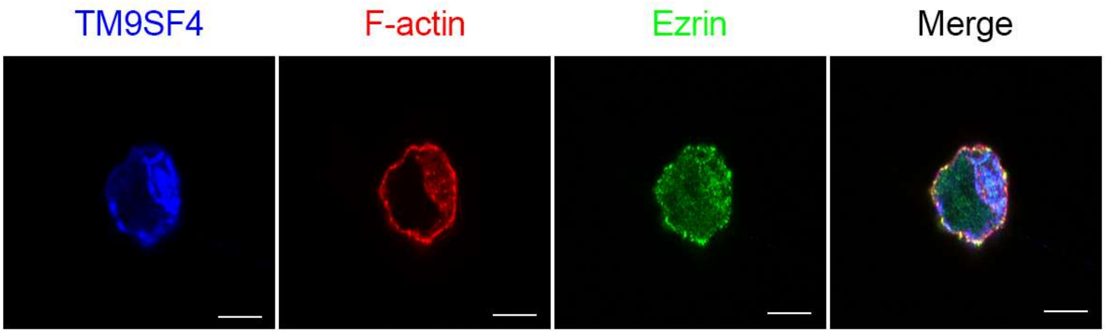

Fig. S18 | Colocalization of TM9SF4, actin and ezrin at cell peripheral regions in A2780 cells. The staining was performed 1 h after cell seeding. Actin was monitored by Rhodamine Phalloidin staining. TM9SF4 and ezrin were monitored by respective antibodies. The spots with white color represent where three proteins are colocalized. Scale bar = 10 μm. The experiments were repeated three times with similar results.

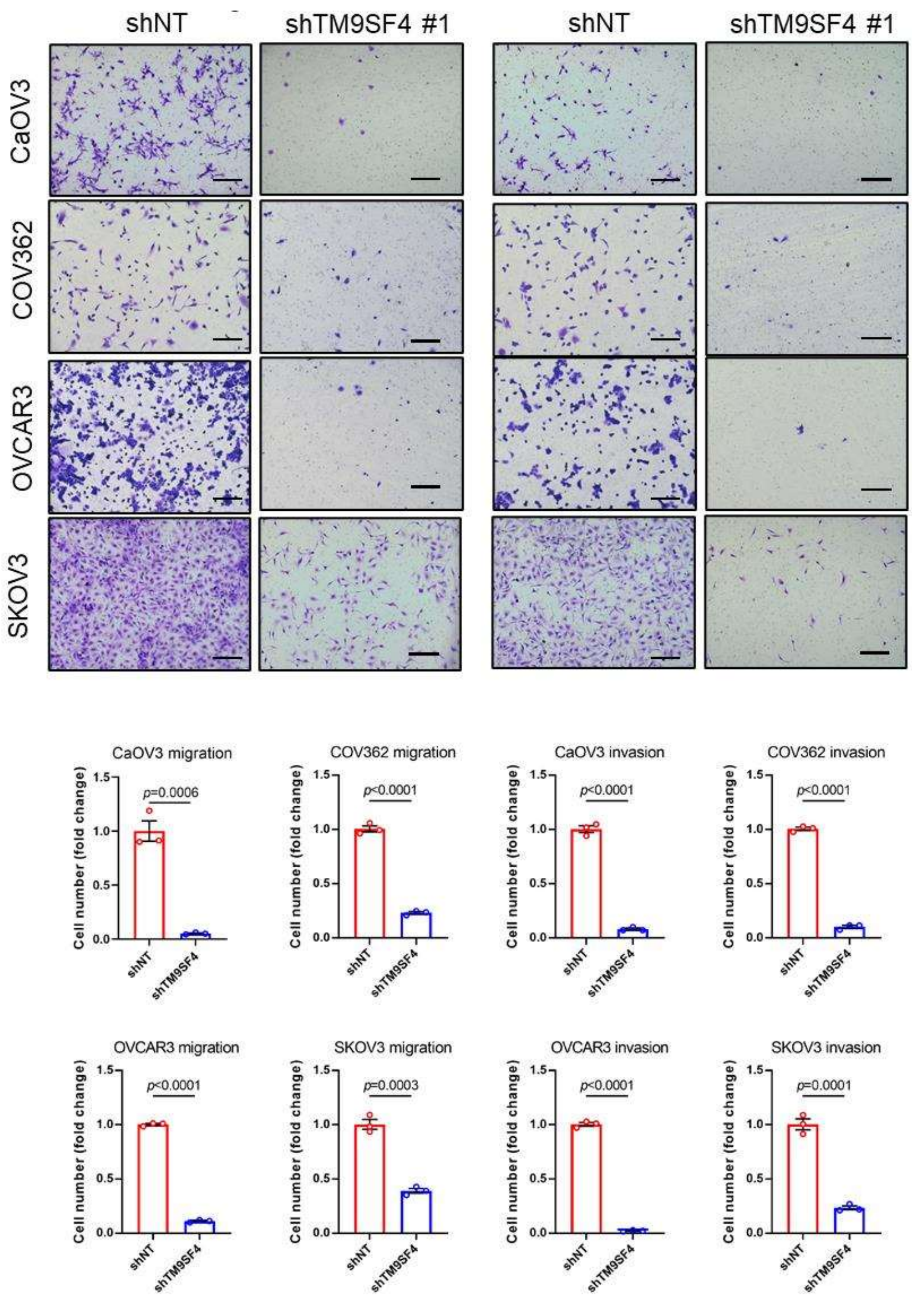

Fig. S19 | TM9SF4 knockdown inhibits the migration and invasion of HGSOCs. Shown are representative images of migration and invasion assays (upper) and summary data (lower). Migration examined the number of cells that transversed the transwell membrane in 24 h. Invasion measured the cells transversed the transwell membrane pre-coated with Matrigel in 24 h. Scale bar = 200 μm. Data are presented as mean ± SEM from 3 biologically independent experiments and two-tailed unpaired Student's t-test was used for statistical analysis. Source data are provided as a Source Data file.

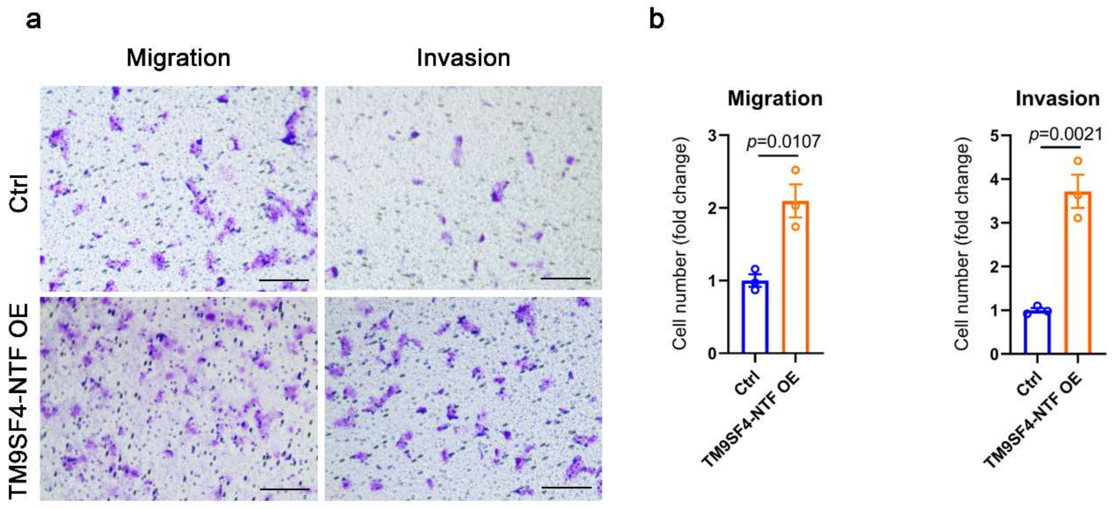

Fig. S20 | NIH3T3 cells that overexpress TM9SF4-NTF show increase in migration and invasion. Migration and invasion assay in NIH3T3 cells expressing an empty vector or TM9SF4-NTF were carried out in transwell chambers. Shown are representative images of migration and invasion assays (**a**) and summary data (**b**). Migration examined the number of NIH3T3 cells that transversed the transwell membrane in 24 h. Invasion measured the cells transversed the transwell membrane pre-coated with Matrigel. Scale bar = 100 µm. Data are presented as mean ± SEM from 3 biologically independent experiments and two-tailed unpaired Student's t-test was used for statistical analysis. Source data are provided as a Source Data file.

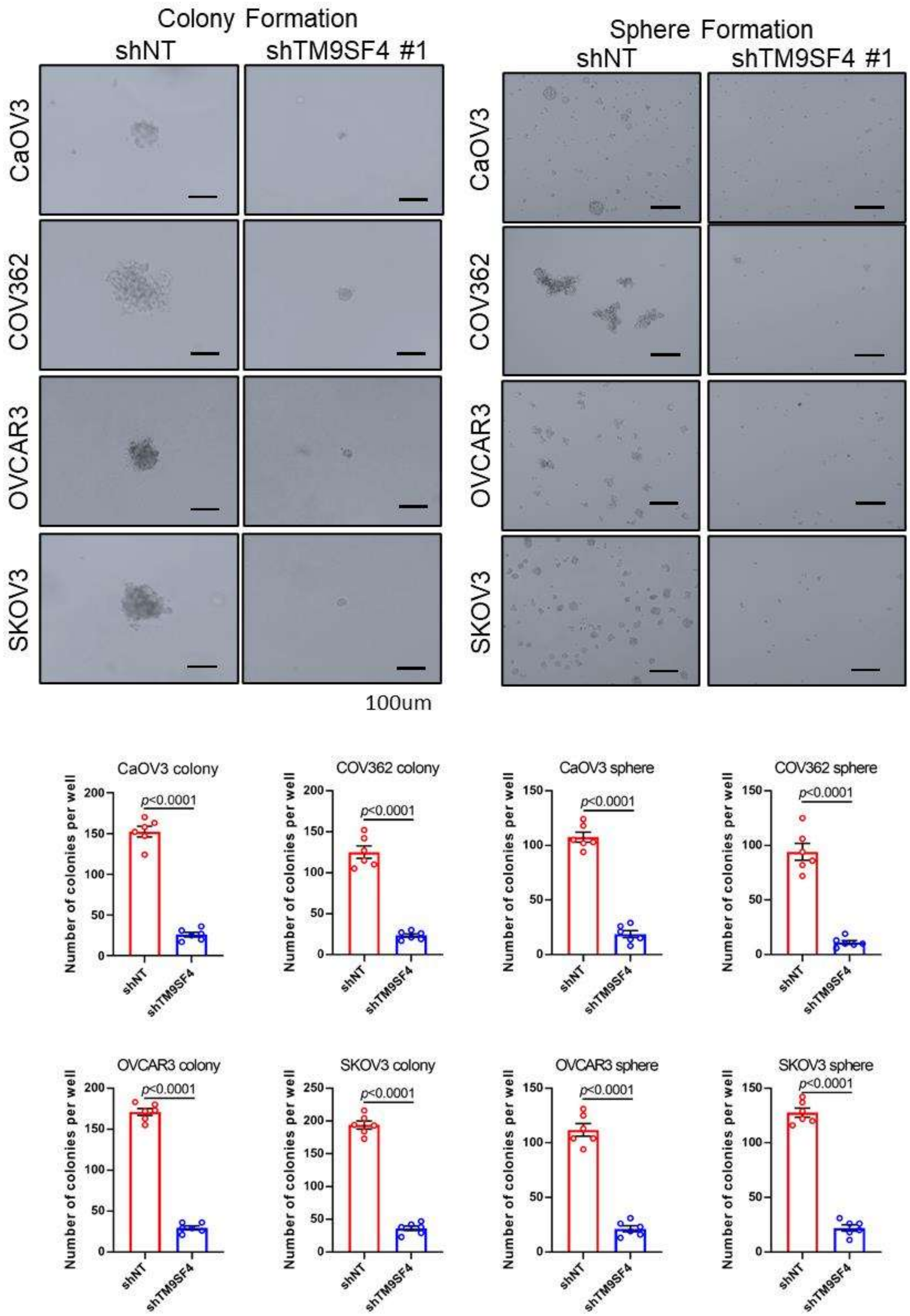

Fig. S21 | TM9SF4 knockdown reduces colony formation and sphere formation of HGSOCs. Shown are representative images of colony formation in soft agar and sphere formation in serum-free stem cell medium (left) and summary data (right). Scale bar = 200 µm. Data are presented as mean ± SEM from 6 biologically independent experiments and two-tailed unpaired Student's t-test was used for statistical analysis. Source data are provided as a Source Data file.

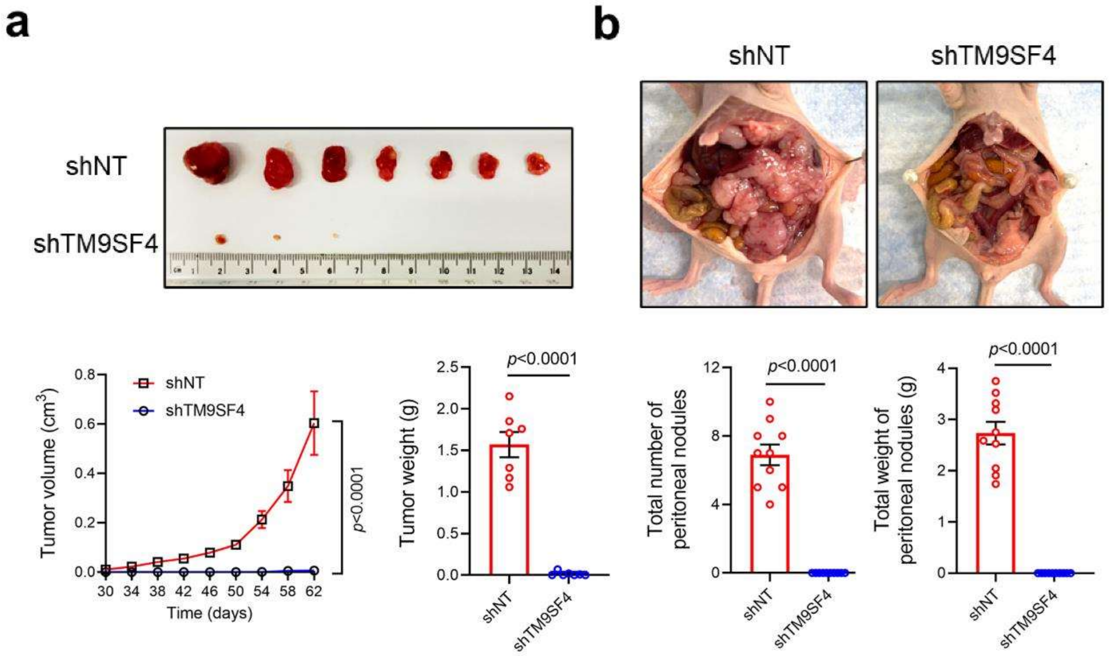

Fig. S22 | TM9SF4 depletion suppresses tumor initiation/growth and metastasis of CaOV3 cells in athymic nude mice. a TM9SF4 knockdown inhibited tumor initiation/growth. Control or TM9SF4 knockdown CaOV3 cells were injected subcutaneously into the hind leg of nude mice, followed by measurement of tumor sizes every 4 days. Shown are representative images of mice (top) and their corresponding dissected tumors (middle), and summary data (bottom) (n = 7 per group). **b** TM9SF4 knockdown inhibited tumor metastasis to peritoneal cavity. Control or TM9SF4 knockdown CaOV3 cells were injected intraperitoneally, followed by measurement of peritoneal tumor nodules in number and weight on day 30 (n = 10 per group). Shown are representative images of peritoneal cavity with tumor nodules (top) and summary data (bottom). Data are presented as mean ± SEM and two-tailed unpaired Student's t-test was used for statistical analysis. Source data are provided as a Source Data file.
