# Supplementary material for: TM9SF4 is an F-actin disassembly factor that promotes tumor progression and metastasis
Source: Nat Commun. 2022 Sep 29;13:5728. doi: 10.1038/s41467-022-33276-y (PMC9522921; doi:10.1038/s41467-022-33276-y)
Supplement: Supplementary file 3 — Description of Additional Supplementary Files [file 41467_2022_33276_MOESM3_ESM.pdf]

Description of Additional Supplementary Files

File Name: Supplementary Data 1
Description: Mass spectrometry data (output of the identification software)
