# Supplementary material for: TM9SF4 is an F-actin disassembly factor that promotes tumor progression and metastasis
Source: Nat Commun. 2022 Sep 29;13:5728. doi: 10.1038/s41467-022-33276-y (PMC9522921; doi:10.1038/s41467-022-33276-y)
Supplement: Supplementary file 4 — Supplementary Data 1 [file 41467_2022_33276_MOESM4_ESM.pdf]

Mascot Search Results

```
User           :
Email          :
Search title   : CAROL
Database       : SwissProt 20100723 (518415 sequences; 182829264 residues)
Taxonomy       : Homo sapiens (human) (20364 sequences)
Timestamp      : 4 Apr 2014 at 08:28:02 GMT
Protein hits   : ACTBM_HUMAN Putative beta-actin-like protein 3 OS=Homo sapiens GN=POTEKP PE=5 SV=1
                 ALBU_HUMAN  Serum albumin OS=Homo sapiens GN=ALB PE=1 SV=2
```

	SwissProt	Decoy	False discovery rate
Peptide matches above identity threshold	3	0	0.00 %
Peptide matches above homology or identity threshold	3	0	0.00 %

Probability Based Mowse Score

Ions score is -10*Log(P), where P is the probability that the observed match is a random event.
Individual ions scores > 30 indicate identity or extensive homology (p<0.05).
Protein scores are derived from ions scores as a non-probabilistic basis for ranking protein hits.

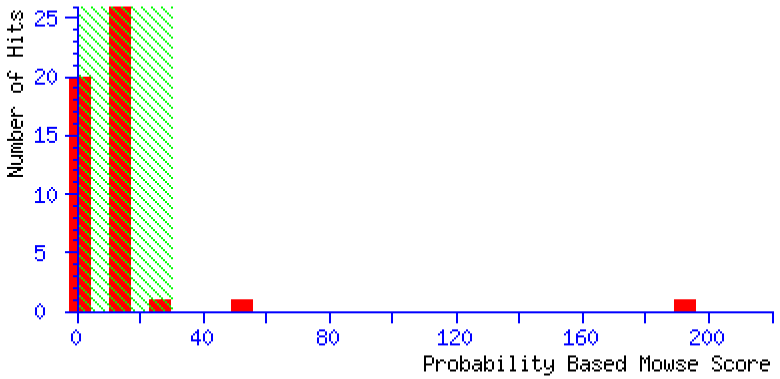

Peptide Summary Report

Format As | Peptide Summary | Help

Significance threshold p< 0.05 | Max. number of hits AUTO

Standard scoring (•) | MudPIT scoring ( ) | Ions score or expect cut-off 0 | Show sub-sets 0

Show pop-ups ◉ Suppress pop-ups ○ Sort unassigned [Decreasing Score ▾] Require bold red ☐

Overview Table

Click on column header to jump to entry in results list.
Move mouse over any indicator to highlight identical peptides.
Click on an indicator to see details of individual match.
Use check boxes to select sub-set of queries for new search.

Mouse over: -Query-
-Accession- -Sequence-

**Hit:**	1	2
☑ 721.3317 (1+)		
☑ 745.3729 (1+)		
☑ 757.3871 (1+)		
☑ 795.4639 (1+)		
☑ 797.3507 (1+)		
☑ 830.4503 (1+)		
☑ 856.5318 (1+)		
☑ 870.5481 (1+)		
☑ 927.5152 (1+)		●
☑ 945.5865 (1+)		
☑ 976.4924 (1+)		
☑ 1046.5936 (1+)		
☑ 1132.5839 (1+)		
☑ 1137.5974 (1+)		
☑ 1163.6849 (1+)		
☑ 1223.4423 (1+)		
☑ 1235.6586 (1+)		
☑ 1249.6912 (1+)		
☑ 1252.6616 (1+)		

1267.8115 (1+)		
1296.7493 (1+)		
1305.7595 (1+)		
1308.7046 (1+)		
1347.7935 (1+)		
1479.8611 (1+)		
1499.7517 (1+)		
1502.2439 (1+)		
1507.8034 (1+)		
1511.9185 (1+)		
1516.7568 (1+)	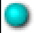	
1537.8616 (1+)		
1547.7975 (1+)		
1567.8135 (1+)		
1619.8699 (1+)		
1639.9921 (1+)		
1759.9875 (1+)		
1772.9887 (1+)		
1790.9576 (1+)	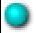	
1823.9721 (1+)		
1868.2588 (1+)		
1899.9205 (1+)		
1940.8635 (1+)		
1954.0683 (1+)		
1960.9522 (1+)		
1976.9675 (1+)		
1983.8199 (1+)		
1992.0512 (1+)		
2008.9162 (1+)		
2093.0942 (1+)		

☑ 2176.2000 (1+)		
☑ 2249.0099 (1+)		
☑ 2586.9908 (1+)		
☑ 3265.1893 (1+)		

Select All | Select None | Search Selected | ☐ **Error tolerant** | Archive Report

1. ACTBM_HUMAN **Mass:** 42331 **Score:** 193 **Queries matched:** 2
Putative beta-actin-like protein 3 OS=Homo sapiens GN=POTEKP PE=5 SV=1
☐ Check to include this hit in error tolerant search or archive report

Query	Observed	Mr(expt)	Mr(calc)	ppm	Miss	Score	Expect	Rank	Peptide
30	1516.7568	1515.7495	1515.6954	35.7	0	88	1e-007	1	K.QEYDESGPSIVHR.K
38	1790.9576	1789.9503	1789.8846	36.7	0	104	2.1e-009	1	K.SYELPDGQVITIGNER.F

Proteins matching the same set of peptides:
ACTB_HUMAN **Mass:** 42052 **Score:** 193 **Queries matched:** 2
Actin, cytoplasmic 1 OS=Homo sapiens GN=ACTB PE=1 SV=1
ACTG_HUMAN **Mass:** 42108 **Score:** 193 **Queries matched:** 2
Actin, cytoplasmic 2 OS=Homo sapiens GN=ACTG1 PE=1 SV=1
POTEE_HUMAN **Mass:** 122882 **Score:** 193 **Queries matched:** 2
POTE ankyrin domain family member E OS=Homo sapiens GN=POTEE PE=1 SV=3
POTEF_HUMAN **Mass:** 123020 **Score:** 193 **Queries matched:** 2
POTE ankyrin domain family member F OS=Homo sapiens GN=POTEF PE=1 SV=2

---

2. ALBU_HUMAN **Mass:** 71317 **Score:** 53 **Queries matched:** 1
Serum albumin OS=Homo sapiens GN=ALB PE=1 SV=2
☐ Check to include this hit in error tolerant search or archive report

Query	Observed	Mr(expt)	Mr(calc)	ppm	Miss	Score	Expect	Rank	Peptide
9	927.5152	926.5079	926.4861	23.5	0	53	0.00028	1	K.YLYEIAR.R

Peptide matches not assigned to protein hits: (no details means no match)

	Query	Observed	Mr(expt)	Mr(calc)	ppm	Miss	Score	Expect	Rank	Peptide
☑	25	1479.8611	1478.8538	1478.7841	47.1	2	13	2.3	1	LADKTDHKGELPR
☑	33	1567.8135	1566.8062	1566.7494	36.3	1	10	5.9	1	MISESGSRMDVLAR + Oxidation (M)
☑	1	*721.3317*	*720.3244*							
☑	2	*745.3729*	*744.3656*							

☑	3	757.3871	756.3798
☑	4	795.4639	794.4566
☑	5	797.3507	796.3435
☑	6	830.4503	829.4430
☑	7	856.5318	855.5245
☑	8	870.5481	869.5409
☑	10	945.5865	944.5792
☑	11	976.4924	975.4851
☑	12	1046.5936	1045.5863
☑	13	1132.5839	1131.5766
☑	14	1137.5974	1136.5901
☑	15	1163.6849	1162.6776
☑	16	1223.4423	1222.4350
☑	17	1235.6586	1234.6513
☑	18	1249.6912	1248.6839
☑	19	1252.6616	1251.6543
☑	20	1267.8115	1266.8042
☑	21	1296.7493	1295.7420
☑	22	1305.7595	1304.7522
☑	23	1308.7046	1307.6973
☑	24	1347.7935	1346.7862
☑	26	1499.7517	1498.7444
☑	27	1502.2439	1501.2366
☑	28	1507.8034	1506.7962
☑	29	1511.9185	1510.9112
☑	31	1537.8616	1536.8543
☑	32	1547.7975	1546.7902
☑	34	1619.8699	1618.8626
☑	35	1639.9921	1638.9848
☑	36	1759.9875	1758.9803
☑	37	1772.9887	1771.9814
☑	39	1823.9721	1822.9648
☑	40	1868.2588	1867.2515
☑	41	1899.9205	1898.9133
☑	42	1940.8635	1939.8563
☑	43	1954.0683	1953.0610

☑	44	*1960.9522*	*1959.9449*
☑	45	*1976.9675*	*1975.9602*
☑	46	*1983.8199*	*1982.8126*
☑	47	*1992.0512*	*1991.0440*
☑	48	*2008.9162*	*2007.9090*
☑	49	*2093.0942*	*2092.0869*
☑	50	*2176.2000*	*2175.1927*
☑	51	*2249.0099*	*2248.0026*
☑	52	*2586.9908*	*2585.9835*
☑	53	*3265.1893*	*3264.1820*

Search Parameters

```
Type of search         : MS/MS Ion Search
Enzyme                 : Trypsin
Fixed modifications    : Carbamidomethyl (C)
Variable modifications : Oxidation (M)
Mass values            : Monoisotopic
Protein Mass           : Unrestricted
Peptide Mass Tolerance : ± 100 ppm
Fragment Mass Tolerance: ± 0.5 Da
Max Missed Cleavages   : 2
Instrument type        : MALDI-TOF-TOF
Number of queries      : 53
```

Mascot Search Results

Protein View

Match to: **ACTBM_HUMAN** Score: **193**
Putative beta-actin-like protein 3 OS=Homo sapiens GN=POTEKP PE=5 SV=1

Nominal mass (M_r): **42331**; Calculated pI value: **5.91**
NCBI BLAST search of ACTBM_HUMAN against nr
Unformatted sequence string for pasting into other applications

Taxonomy: Homo sapiens

Fixed modifications: Carbamidomethyl (C)
Variable modifications: Oxidation (M)
Cleavage by Trypsin: cuts C-term side of KR unless next residue is P
Sequence Coverage: **7%**

Matched peptides shown in **Bold Red**

```
  1 MDDDTAVLVI DNGSGMCKAG FAGDDAPQAV FPSIVGRPRH QGMMEGMHQK
 51 ESYVGKEAQS KRGMLTLKYP MEHGIITNWD DMEKIWHHTF YNELRVAPEE
101 HPILLTEAPL NPKANREKMT QIMFETFNTP AMYVAIQAVL SLYTSGRTTG
151 IVMDSGDGFT HTVPIYEGNA LPHATLRLDL AGRELTDYLM KILTERGYRF
201 TTTAEQEIVR DIKEKLCYVA LDSEQEMAMA ASSSSVEKSY ELPDGQVITI
251 GNERFRCPEA LFQPCFLGME SCGIHKTTFN SIVKSDVDIR KDLYTNTVLS
301 GGTTMYPGIA HRMQKEITAL APSIMKIKII APPKRKYSVW VGGSILASLS
351 TFQQMWISKQ EYDESGPSIV HRKCF
```

Show predicted peptides also

Sort Peptides By ◉ Residue Number ○ Increasing Mass ○ Decreasing Mass

Start - End	Observed	Mr(expt)	Mr(calc)	ppm	Miss	Sequence
239 - 254	1790.9576	1789.9503	1789.8846	37	0	K.SYELPDGQVITIGNER.F (Ions score 104)
360 - 372	1516.7568	1515.7495	1515.6954	36	0	K.QEYDESGPSIVHR.K (Ions score 88)

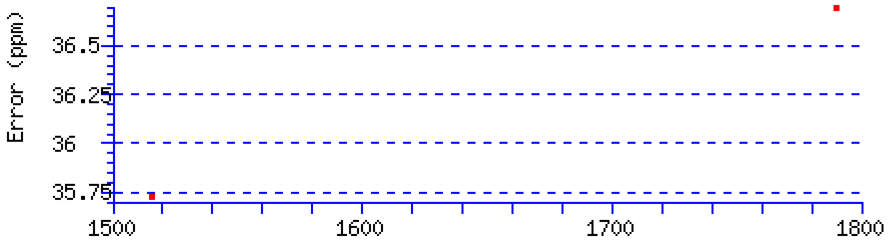

RMS error 36 ppm

```
ID   ACTBM_HUMAN             Reviewed;         375 AA.
AC   Q9BYX7; Q562N5;
DT   23-OCT-2007, integrated into UniProtKB/Swiss-Prot.
DT   01-JUN-2001, sequence version 1.
DT   13-JUL-2010, entry version 51.
DE   RecName: Full=Putative beta-actin-like protein 3;
DE   AltName: Full=POTE ankyrin domain family member K;
DE   AltName: Full=Kappa-actin;
GN   Name=POTEKP; Synonyms=ACTBL3; ORFNames=FKSG30;
OS   Homo sapiens (Human).
OC   Eukaryota; Metazoa; Chordata; Craniata; Vertebrata; Euteleostomi;
OC   Mammalia; Eutheria; Euarchontoglires; Primates; Haplorrhini;
OC   Catarrhini; Hominidae; Homo.
OX   NCBI_TaxID=9606;
RN   [1]
RP   NUCLEOTIDE SEQUENCE [MRNA].
RA   Wang Y.-G., Gong L.;
RT   "Cloning and characterization of FKSG30, a novel gene encoding a
RT   protein similar to ACTG1.";
RL   Submitted (NOV-2000) to the EMBL/GenBank/DDBJ databases.
RN   [2]
RP   NUCLEOTIDE SEQUENCE [GENOMIC DNA] OF 84-186, INTERACTION WITH PFN1 AND
RP   PFDN1, AND TISSUE SPECIFICITY.
RC   TISSUE=Liver;
RX   PubMed=16824795; DOI=10.1016/j.hepres.2006.05.003;
RA   Chang K.-W., Yang P.-Y., Lai H.-Y., Yeh T.-S., Chen T.-C., Yeh C.-T.;
RT   "Identification of a novel actin isoform in hepatocellular
RT   carcinoma.";
RL   Hepatol. Res. 36:33-39(2006).
CC   -!- SUBUNIT: Interacts with PFN1 and PFDN1. Does not interact with
CC       PFN2.
CC   -!- SUBCELLULAR LOCATION: Cytoplasm, cytoskeleton (By similarity).
CC   -!- TISSUE SPECIFICITY: Expressed in some hepatocellular carcinomas.
CC   -!- SIMILARITY: Belongs to the actin family.
CC   -!- CAUTION: Could be the product of a pseudogene.
CC   -----------------------------------------------------------------------
CC   Copyrighted by the UniProt Consortium, see http://www.uniprot.org/terms
CC   Distributed under the Creative Commons Attribution-NoDerivs License
CC   -----------------------------------------------------------------------
DR   EMBL; AY014272; AAG50355.1; -; mRNA.
DR   EMBL; AY970480; AAX82286.1; -; Genomic_DNA.
DR   IPI; IPI00888712; -.
DR   UniGene; Hs.631267; -.
DR   UniGene; Hs.654289; -.
DR   HSSP; P60712; 2BTF.
DR   SMR; Q9BYX7; 2-375.
DR   IntAct; Q9BYX7; 3.
DR   MINT; MINT-1141007; -.
DR   STRING; Q9BYX7; -.
DR   PhosphoSite; Q9BYX7; -.
DR   PRIDE; Q9BYX7; -.
DR   Ensembl; ENST00000451531; ENSP00000392718; ENSG00000196834; Homo sapiens.
```

```
DR   UCSC; uc010fmg.1; human.
DR   GeneCards; GC02M131217; -.
DR   GeneCards; GC02M131218; -.
DR   H-InvDB; HIX0024222; -.
DR   H-InvDB; HIX0037703; -.
DR   HGNC; HGNC:30182; POTEKP.
DR   MIM; 611266; gene.
DR   HOGENOM; HBG506345; -.
DR   PhylomeDB; Q9BYX7; -.
DR   Reactome; REACT_13527; Further platelet releasate.
DR   Reactome; REACT_604; Hemostasis.
DR   Bgee; Q9BYX7; -.
DR   Genevestigator; Q9BYX7; -.
DR   GO; GO:0005856; C:cytoskeleton; IEA:UniProtKB-SubCell.
DR   GO; GO:0005829; C:cytosol; EXP:Reactome.
DR   GO; GO:0005576; C:extracellular region; EXP:Reactome.
DR   GO; GO:0005524; F:ATP binding; IEA:UniProtKB-KW.
DR   GO; GO:0005515; F:protein binding; IEA:InterPro.
DR   InterPro; IPR004000; Actin-like.
DR   InterPro; IPR020902; Actin/actin-like_CS.
DR   InterPro; IPR004001; Actin_CS.
DR   PANTHER; PTHR11937; Actin_like; 1.
DR   Pfam; PF00022; Actin; 1.
DR   PRINTS; PR00190; ACTIN.
DR   SMART; SM00268; ACTIN; 1.
DR   PROSITE; PS00432; ACTINS_2; 1.
DR   PROSITE; PS01132; ACTINS_ACT_LIKE; 1.
PE   5: Uncertain;
KW   ATP-binding; Complete proteome; Cytoplasm; Cytoskeleton;
KW   Nucleotide-binding.
FT   CHAIN         1    375       Putative beta-actin-like protein 3.
FT                                /FTId=PRO_0000307865.
FT   CONFLICT    159    159       F -> V (in Ref. 2; AAX82286).
FT   CONFLICT    177    177       R -> P (in Ref. 2; AAX82286).
SQ   SEQUENCE   375 AA;  42016 MW;  98127B88A9983B7D CRC64;
     MDDDTAVLVI DNGSGMCKAG FAGDDAPQAV FPSIVGRPRH QGMMEGMHQK ESYVGKEAQS
     KRGMLTLKYP MEHGIITNWD DMEKIWHHTF YNELRVAPEE HPILLTEAPL NPKANREKMT
     QIMFETFNTP AMYVAIQAVL SLYTSGRTTG IVMDSGDGFT HTVPIYEGNA LPHATLRLDL
     AGRELTDYLM KILTERGYRF TTTAEQEIVR DIKEKLCYVA LDSEQEMAMA ASSSSVEKSY
     ELPDGQVITI GNERFRCPEA LFQPCFLGME SCGIHKTTFN SIVKSDVDIR KDLYTNTVLS
     GGTTMYPGIA HRMQKEITAL APSIMKIKII APPKRKYSVW VGGSILASLS TFQQMWISKQ
     EYDESGPSIV HRKCF
```

Mascot Search Results

Protein View

Match to: **ACTB_HUMAN** Score: **62** Expect: **0.014**
Actin, cytoplasmic 1 OS=Homo sapiens GN=ACTB PE=1 SV=1

Nominal mass (M_r): **42052**; Calculated pI value: **5.29**
NCBI BLAST search of ACTB_HUMAN against nr
Unformatted sequence string for pasting into other applications

Taxonomy: Homo sapiens

Fixed modifications: Carbamidomethyl (C)
Variable modifications: Oxidation (M)
Cleavage by Trypsin: cuts C-term side of KR unless next residue is P
Number of mass values searched: **53**
Number of mass values matched: **7**
Sequence Coverage: **23%**

Matched peptides shown in **Bold Red**

```
  1 MDDDIAALVV DNGSGMCKAG FAGDDAPRAV FPSIVGRPRH QGVMVGMGQK
 51 DSYVGDEAQS KRGILTLKYP IEHGIVTNWD DMEKIWHHTF YNELRVAPEE
101 HPVLLTEAPL NPKANREKMT QIMFETFNTP AMYVAIQAVL SLYASGRTTG
151 IVMDSGDGVT HTVPIYEGYA LPHAILRLDL AGRDLTDYLM KILTERGYSF
201 TTTAEREIVR DIKEKLCYVA LDFEQEMATA ASSSSLEKSY ELPDGQVITI
251 GNERFRCPEA LFQPSFLGME SCGIHETTFN SIMKCDVDIR KDLYANTVLS
301 GGTTMYPGIA DRMQKEITAL APSTMKIKII APPERKYSVW IGGSILASLS
351 TFQQMWISKQ EYDESGPSIV HRKCF
```

Show predicted peptides also

Sort Peptides By ○ Residue Number ● Increasing Mass ○ Decreasing Mass

Start - End	Observed	Mr(expt)	Mr(calc)	ppm	Miss	Sequence
329 - 335	795.4639	794.4566	794.4650	-11	0	K.IIAPPER.K
19 - 28	976.4924	975.4851	975.4410	45	0	K.AGFAGDDAPR.A
197 - 206	1132.5839	1131.5766	1131.5197	50	0	R.GYSFTTTAER.E
360 - 372	1516.7568	1515.7495	1515.6954	36	0	K.QEYDESGPSIVHR.K
178 - 191	1639.9921	1638.9848	1638.8287	95	1	R.LDLAGRDLTDYLMK.I Oxidation (M)
239 - 254	1790.9576	1789.9503	1789.8846	37	0	K.SYELPDGQVITIGNER.F
96 - 113	1954.0683	1953.0610	1953.0571	2	0	R.VAPEEHPVLLTEAPLNPK.A

No match to: 721.3317, 745.3729, 757.3871, 797.3507, 830.4503, 856.5318, 870.5481, 927.5152, 945.5865, 1046.5936, 1137.5974, 1163.6849, 1223.4423, 123[illegible]

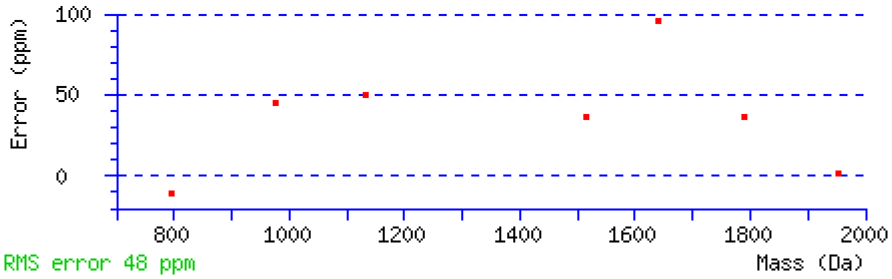


```
ID   ACTB_HUMAN              Reviewed;         375 AA.
AC   P60709; P02570; P70514; P99021; Q11211; Q64316; Q75MN2; Q96B34;
AC   Q96HG5;
DT   21-JUL-1986, integrated into UniProtKB/Swiss-Prot.
DT   01-APR-1988, sequence version 1.
DT   13-JUL-2010, entry version 88.
DE   RecName: Full=Actin, cytoplasmic 1;
DE   AltName: Full=Beta-actin;
DE   Contains:
DE     RecName: Full=Actin, cytoplasmic 1, N-terminally processed;
GN   Name=ACTB;
OS   Homo sapiens (Human).
OC   Eukaryota; Metazoa; Chordata; Craniata; Vertebrata; Euteleostomi;
OC   Mammalia; Eutheria; Euarchontoglires; Primates; Haplorrhini;
OC   Catarrhini; Hominidae; Homo.
OX   NCBI_TaxID=9606;
RN   [1]
RP   NUCLEOTIDE SEQUENCE [MRNA].
RX   MEDLINE=84144061; PubMed=6322116; DOI=10.1093/nar/12.3.1687;
RA   Ponte P., Ng S.Y., Engel J., Gunning P., Kedes L.;
RT   "Evolutionary conservation in the untranslated regions of actin mRNAs:
RT   DNA sequence of a human beta-actin cDNA.";
RL   Nucleic Acids Res. 12:1687-1696(1984).
RN   [2]
RP   NUCLEOTIDE SEQUENCE [GENOMIC DNA].
RX   MEDLINE=85298307; PubMed=2994062; DOI=10.1073/pnas.82.18.6133;
RA   Nakajima-Iijima S., Hamada H., Reddy P., Kakunaga T.;
RT   "Molecular structure of the human cytoplasmic beta-actin gene:
RT   interspecies homology of sequences in the introns.";
RL   Proc. Natl. Acad. Sci. U.S.A. 82:6133-6137(1985).
RN   [3]
RP   NUCLEOTIDE SEQUENCE [MRNA].
RX   PubMed=1734024; DOI=10.1083/jcb.116.4.933;
RA   Ohmori H., Toyama S., Toyama S.;
RT   "Direct proof that the primary site of action of cytochalasin on cell
RT   motility processes is actin.";
RL   J. Cell Biol. 116:933-941(1992).
RN   [4]
RP   NUCLEOTIDE SEQUENCE [GENOMIC DNA].
```

```
RG   NIEHS SNPs program;
RL   Submitted (MAR-2004) to the EMBL/GenBank/DDBJ databases.
RN   [5]
RP   NUCLEOTIDE SEQUENCE [LARGE SCALE GENOMIC DNA].
RX   MEDLINE=22737999; PubMed=12853948; DOI=10.1038/nature01782;
RA   Hillier L.W., Fulton R.S., Fulton L.A., Graves T.A., Pepin K.H.,
RA   Wagner-McPherson C., Layman D., Maas J., Jaeger S., Walker R.,
RA   Wylie K., Sekhon M., Becker M.C., O'Laughlin M.D., Schaller M.E.,
RA   Fewell G.A., Delehaunty K.D., Miner T.L., Nash W.E., Cordes M., Du H.,
RA   Sun H., Edwards J., Bradshaw-Cordum H., Ali J., Andrews S., Isak A.,
RA   Vanbrunt A., Nguyen C., Du F., Lamar B., Courtney L., Kalicki J.,
RA   Ozersky P., Bielicki L., Scott K., Holmes A., Harkins R., Harris A.,
RA   Strong C.M., Hou S., Tomlinson C., Dauphin-Kohlberg S.,
RA   Kozlowicz-Reilly A., Leonard S., Rohlfing T., Rock S.M.,
RA   Tin-Wollam A.-M., Abbott A., Minx P., Maupin R., Strowmatt C.,
RA   Latreille P., Miller N., Johnson D., Murray J., Woessner J.P.,
RA   Wendl M.C., Yang S.-P., Schultz B.R., Wallis J.W., Spieth J.,
RA   Bieri T.A., Nelson J.O., Berkowicz N., Wohldmann P.E., Cook L.L.,
RA   Hickenbotham M.T., Eldred J., Williams D., Bedell J.A., Mardis E.R.,
RA   Clifton S.W., Chissoe S.L., Marra M.A., Raymond C., Haugen E.,
RA   Gillett W., Zhou Y., James R., Phelps K., Iadanoto S., Bubb K.,
RA   Simms E., Levy R., Clendenning J., Kaul R., Kent W.J., Furey T.S.,
RA   Baertsch R.A., Brent M.R., Keibler E., Flicek P., Bork P., Suyama M.,
RA   Bailey J.A., Portnoy M.E., Torrents D., Chinwalla A.T., Gish W.R.,
RA   Eddy S.R., McPherson J.D., Olson M.V., Eichler E.E., Green E.D.,
RA   Waterston R.H., Wilson R.K.;
RT   "The DNA sequence of human chromosome 7.";
RL   Nature 424:157-164(2003).
RN   [6]
RP   NUCLEOTIDE SEQUENCE [LARGE SCALE MRNA].
RC   TISSUE=Brain, Eye, Kidney, Muscle, Pancreas, Placenta, and Skin;
RX   PubMed=15489334; DOI=10.1101/gr.2596504;
RG   The MGC Project Team;
RT   "The status, quality, and expansion of the NIH full-length cDNA
RT   project: the Mammalian Gene Collection (MGC).";
RL   Genome Res. 14:2121-2127(2004).
RN   [7]
RP   PROTEIN SEQUENCE OF 2-28.
RC   TISSUE=Platelet;
RX   MEDLINE=22608298; PubMed=12665801; DOI=10.1038/nbt810;
RA   Gevaert K., Goethals M., Martens L., Van Damme J., Staes A.,
RA   Thomas G.R., Vandekerckhove J.;
RT   "Exploring proteomes and analyzing protein processing by mass
RT   spectrometric identification of sorted N-terminal peptides.";
RL   Nat. Biotechnol. 21:566-569(2003).
RN   [8]
RP   PROTEIN SEQUENCE OF 2-18; 29-37; 40-50; 85-95; 148-177; 184-191;
RP   197-206; 292-312 AND 316-326, CLEAVAGE OF INITIATOR METHIONINE,
RP   ACETYLATION AT ASP-2, AND MASS SPECTROMETRY.
RC   TISSUE=B-cell lymphoma;
RA   Bienvenut W.V.;
RL   Submitted (JUN-2005) to UniProtKB.
RN   [9]
```

```
RP   PROTEIN SEQUENCE OF 19-62; 85-113; 184-191; 197-206; 216-254; 291-312;
RP   316-326 AND 360-372, AND MASS SPECTROMETRY.
RC   TISSUE=Brain, Cajal-Retzius cell, and Fetal brain cortex;
RA   Lubec G., Afjehi-Sadat L., Chen W.-Q., Sun Y.;
RL   Submitted (DEC-2008) to UniProtKB.
RN   [10]
RP   NUCLEOTIDE SEQUENCE [MRNA] OF 252-375.
RX   MEDLINE=83189093; PubMed=6842590; DOI=10.1016/0022-2836(83)90117-1;
RA   Hanukoglu I., Tanese N., Fuchs E.;
RT   "Complementary DNA sequence of a human cytoplasmic actin. Interspecies
RT   divergence of 3' non-coding regions.";
RL   J. Mol. Biol. 163:673-678(1983).
RN   [11]
RP   IDENTIFICATION IN A COMPLEX WITH RAN; XPO6 AND PFN1, AND INTERACTION
RP   WITH XPO6.
RX   PubMed=14592989; DOI=10.1093/emboj/cdg565;
RA   Stueven T., Hartmann E., Goerlich D.;
RT   "Exportin 6: a novel nuclear export receptor that is specific for
RT   profilin.actin complexes.";
RL   EMBO J. 22:5928-5940(2003).
RN   [12]
RP   INTERACTION WITH EMD.
RX   PubMed=15328537; DOI=10.1371/journal.pbio.0020231;
RA   Holaska J.M., Kowalski A.K., Wilson K.L.;
RT   "Emerin caps the pointed end of actin filaments: evidence for an actin
RT   cortical network at the nuclear inner membrane.";
RL   PLoS Biol. 2:1354-1362(2004).
RN   [13]
RP   PHOSPHORYLATION [LARGE SCALE ANALYSIS] AT TYR-166; TYR-218 AND
RP   TYR-294, AND MASS SPECTROMETRY.
RX   PubMed=15592455; DOI=10.1038/nbt1046;
RA   Rush J., Moritz A., Lee K.A., Guo A., Goss V.L., Spek E.J., Zhang H.,
RA   Zha X.-M., Polakiewicz R.D., Comb M.J.;
RT   "Immunoaffinity profiling of tyrosine phosphorylation in cancer
RT   cells.";
RL   Nat. Biotechnol. 23:94-101(2005).
RN   [14]
RP   PHOSPHORYLATION [LARGE SCALE ANALYSIS] AT TYR-53; TYR-91; TYR-198 AND
RP   TYR-294, AND MASS SPECTROMETRY.
RC   TISSUE=Lung carcinoma;
RX   PubMed=18083107; DOI=10.1016/j.cell.2007.11.025;
RA   Rikova K., Guo A., Zeng Q., Possemato A., Yu J., Haack H., Nardone J.,
RA   Lee K., Reeves C., Li Y., Hu Y., Tan Z., Stokes M., Sullivan L.,
RA   Mitchell J., Wetzel R., Macneill J., Ren J.M., Yuan J.,
RA   Bakalarski C.E., Villen J., Kornhauser J.M., Smith B., Li D., Zhou X.,
RA   Gygi S.P., Gu T.-L., Polakiewicz R.D., Rush J., Comb M.J.;
RT   "Global survey of phosphotyrosine signaling identifies oncogenic
RT   kinases in lung cancer.";
RL   Cell 131:1190-1203(2007).
RN   [15]
RP   IDENTIFICATION IN A MRNP GRANULE COMPLEX, IDENTIFICATION BY MASS
RP   SPECTROMETRY, AND SUBCELLULAR LOCATION.
RX   PubMed=17289661; DOI=10.1074/mcp.M600346-MCP200;
```

RA   Joeson L., Vikesaa J., Krogh A., Nielsen L.K., Hansen T., Borup R.,
RA   Johnsen A.H., Christiansen J., Nielsen F.C.;
RT   "Molecular composition of IMP1 ribonucleoprotein granules.";
RL   Mol. Cell. Proteomics 6:798-811(2007).
RN   [16]
RP   IDENTIFICATION IN THE BAF COMPLEX, AND IDENTIFICATION BY MASS
RP   SPECTROMETRY.
RX   PubMed=18765789; DOI=10.1101/gad.471408;
RA   Lange M., Kaynak B., Forster U.B., Toenjes M., Fischer J.J., Grimm C.,
RA   Schlesinger J., Just S., Dunkel I., Krueger T., Mebus S., Lehrach H.,
RA   Lurz R., Gobom J., Rottbauer W., Abdelilah-Seyfried S., Sperling S.;
RT   "Regulation of muscle development by DPF3, a novel histone acetylation
RT   and methylation reader of the BAF chromatin remodeling complex.";
RL   Genes Dev. 22:2370-2384(2008).
RN   [17]
RP   PHOSPHORYLATION [LARGE SCALE ANALYSIS] AT THR-318, AND MASS
RP   SPECTROMETRY.
RC   TISSUE=Platelet;
RX   PubMed=18088087; DOI=10.1021/pr0704130;
RA   Zahedi R.P., Lewandrowski U., Wiesner J., Wortelkamp S., Moebius J.,
RA   Schuetz C., Walter U., Gambaryan S., Sickmann A.;
RT   "Phosphoproteome of resting human platelets.";
RL   J. Proteome Res. 7:526-534(2008).
RN   [18]
RP   PHOSPHORYLATION [LARGE SCALE ANALYSIS] AT TYR-53; TYR-91; TYR-169 AND
RP   TYR-198, AND MASS SPECTROMETRY.
RC   TISSUE=Mammary epithelium;
RX   PubMed=19534553; DOI=10.1021/pr900044c;
RA   Heibeck T.H., Ding S.-J., Opresko L.K., Zhao R., Schepmoes A.A.,
RA   Yang F., Tolmachev A.V., Monroe M.E., Camp D.G. II, Smith R.D.,
RA   Wiley H.S., Qian W.-J.;
RT   "An extensive survey of tyrosine phosphorylation revealing new sites
RT   in human mammary epithelial cells.";
RL   J. Proteome Res. 8:3852-3861(2009).
RN   [19]
RP   PHOSPHORYLATION [LARGE SCALE ANALYSIS] AT TYR-169; TYR-198; TYR-218
RP   AND TYR-294, AND MASS SPECTROMETRY.
RC   TISSUE=Leukemic T-cell;
RX   PubMed=19690332; DOI=10.1126/scisignal.2000007;
RA   Mayya V., Lundgren D.H., Hwang S.-I., Rezaul K., Wu L., Eng J.K.,
RA   Rodionov V., Han D.K.;
RT   "Quantitative phosphoproteomic analysis of T cell receptor signaling
RT   reveals system-wide modulation of protein-protein interactions.";
RL   Sci. Signal. 2:RA46-RA46(2009).
RN   [20]
RP   VARIANT DYTJ TRP-183, AND CHARACTERIZATION OF VARIANT DYTJ TRP-183.
RX   PubMed=16685646; DOI=10.1086/504271;
RA   Procaccio V., Salazar G., Ono S., Styers M.L., Gearing M., Davila A.,
RA   Jimenez R., Juncos J., Gutekunst C.-A., Meroni G., Fontanella B.,
RA   Sontag E., Sontag J.-M., Faundez V., Wainer B.H.;
RT   "A mutation of beta -actin that alters depolymerization dynamics is
RT   associated with autosomal dominant developmental malformations,
RT   deafness, and dystonia.";

```
RL   Am. J. Hum. Genet. 78:947-960(2006).
RN   [21]
RP   IDENTIFICATION IN THE MLL5-L COMPLEX.
RX   PubMed=19377461; DOI=10.1038/nature07954;
RA   Fujiki R., Chikanishi T., Hashiba W., Ito H., Takada I., Roeder R.G.,
RA   Kitagawa H., Kato S.;
RT   "GlcNAcylation of a histone methyltransferase in retinoic-acid-induced
RT   granulopoiesis.";
RL   Nature 459:455-459(2009).
CC   -!- FUNCTION: Actins are highly conserved proteins that are involved
CC       in various types of cell motility and are ubiquitously expressed
CC       in all eukaryotic cells.
CC   -!- SUBUNIT: Polymerization of globular actin (G-actin) leads to a
CC       structural filament (F-actin) in the form of a two-stranded helix.
CC       Each actin can bind to 4 others. Identified in a mRNP granule
CC       complex, at least composed of ACTB, ACTN4, DHX9, ERG, HNRNPA1,
CC       HNRNPA2B1, HNRNPAB, HNRNPD, HNRNPL, HNRNPR, HNRNPU, HSPA1, HSPA8,
CC       IGF2BP1, ILF2, ILF3, NCBP1, NCL, PABPC1, PABPC4, PABPN1, RPLP0,
CC       RPS3, RPS3A, RPS4X, RPS8, RPS9, SYNCRIP, TROVE2, YBX1 and
CC       untranslated mRNAs. Component of the BAF complex, which includes
CC       at least actin (ACTB), ARID1A, ARID1B/BAF250, SMARCA2,
CC       SMARCA4/BRG1, ACTL6A/BAF53, ACTL6B/BAF53B, SMARCE1/BAF57
CC       SMARCC1/BAF155, SMARCC2/BAF170, SMARCB1/SNF5/INI1, and one or more
CC       of SMARCD1/BAF60A, SMARCD2/BAF60B, or SMARCD3/BAF60C. In muscle
CC       cells, the BAF complex also contains DPF3. Found in a complex with
CC       XPO6, Ran, ACTB and PFN1. Component of the MLL5-L complex, at
CC       least composed of MLL5, STK38, PPP1CA, PPP1CB, PPP1CC, HCFC1, ACTB
CC       and OGT. Interacts with XPO6 and EMD.
CC   -!- INTERACTION:
CC       Q9Y281:CFL2; NbExp=1; IntAct=EBI-353944, EBI-351218;
CC       Q00987:MDM2; NbExp=1; IntAct=EBI-353944, EBI-389668;
CC       P84022:SMAD3; NbExp=1; IntAct=EBI-353944, EBI-347161;
CC   -!- SUBCELLULAR LOCATION: Cytoplasm, cytoskeleton. Note=Localized in
CC       cytoplasmic mRNP granules containing untranslated mRNAs.
CC   -!- DISEASE: Defects in ACTB are a cause of dystonia juvenile-onset
CC       (DYTJ) [MIM:607371]. DYTJ is a form of dystonia with juvenile
CC       onset. Dystonia is defined by the presence of sustained
CC       involuntary muscle contraction, often leading to abnormal
CC       postures. DYTJ patients manifest progressive, generalized, dopa-
CC       unresponsive dystonia, developmental malformations and sensory
CC       hearing loss.
CC   -!- MISCELLANEOUS: In vertebrates 3 main groups of actin isoforms,
CC       alpha, beta and gamma have been identified. The alpha actins are
CC       found in muscle tissues and are a major constituent of the
CC       contractile apparatus. The beta and gamma actins coexist in most
CC       cell types as components of the cytoskeleton and as mediators of
CC       internal cell motility.
CC   -!- SIMILARITY: Belongs to the actin family.
CC   -!- WEB RESOURCE: Name=Atlas of Genetics and Cytogenetics in Oncology
CC       and Haematology;
CC       URL="http://atlasgeneticsoncology.org/Genes/ACTBID42959ch7p22.html";
CC   -!- WEB RESOURCE: Name=NIEHS-SNPs;
CC       URL="http://egp.gs.washington.edu/data/actb/";
```

CC   ---
CC   Copyrighted by the UniProt Consortium, see http://www.uniprot.org/terms
CC   Distributed under the Creative Commons Attribution-NoDerivs License
CC   ---


```
DR   EMBL; X00351; CAA25099.1; -; mRNA.
DR   EMBL; M10277; AAA51567.1; -; Genomic_DNA.
DR   EMBL; X63432; CAA45026.1; -; mRNA.
DR   EMBL; AY582799; AAS79319.1; -; Genomic_DNA.
DR   EMBL; AC006483; AAP22343.1; -; Genomic_DNA.
DR   EMBL; BC001301; AAH01301.1; -; mRNA.
DR   EMBL; BC002409; AAH02409.1; -; mRNA.
DR   EMBL; BC004251; AAH04251.1; -; mRNA.
DR   EMBL; BC008633; AAH08633.1; -; mRNA.
DR   EMBL; BC012854; AAH12854.1; -; mRNA.
DR   EMBL; BC013380; AAH13380.1; -; mRNA.
DR   EMBL; BC014861; AAH14861.1; -; mRNA.
DR   EMBL; BC016045; AAH16045.1; -; mRNA.
DR   EMBL; V00478; CAA23745.1; -; mRNA.
DR   IPI; IPI00021439; -.
DR   PIR; A25168; ATHUB.
DR   RefSeq; NP_001092.1; -.
DR   UniGene; Hs.520640; -.
DR   PDB; 3BYH; EM; 12.00 A; A=2-375.
DR   PDB; 3D2U; X-ray; 2.21 A; C/G=170-178.
DR   PDB; 3LUE; EM; -; A/B/C/D/E/F/G/H/I/J=2-375.
DR   PDBsum; 3BYH; -.
DR   PDBsum; 3D2U; -.
DR   PDBsum; 3LUE; -.
DR   SMR; P60709; 2-375.
DR   DIP; DIP-29686N; -.
DR   IntAct; P60709; 98.
DR   MINT; MINT-220312; -.
DR   STRING; P60709; -.
DR   PhosphoSite; P60709; -.
DR   SWISS-2DPAGE; P60709; -.
DR   Aarhus/Ghent-2DPAGE; 7316; IEF.
DR   DOSAC-COBS-2DPAGE; P60709; -.
DR   DOSAC-COBS-2DPAGE; P60709_OR_P63261; -.
DR   REPRODUCTION-2DPAGE; P60709; -.
DR   Siena-2DPAGE; P60709; -.
DR   UCD-2DPAGE; P60709; -.
DR   PeptideAtlas; P60709; -.
DR   PRIDE; P60709; -.
DR   Ensembl; ENST00000331789; ENSP00000349960; ENSG00000075624; Homo sapiens.
DR   GeneID; 60; -.
DR   KEGG; hsa:60; -.
DR   UCSC; uc003sos.2; human.
DR   CTD; 60; -.
DR   GeneCards; GC07M005533; -.
DR   H-InvDB; HIX0006454; -.
DR   HGNC; HGNC:132; ACTB.
DR   HPA; CAB002621; -.
DR   MIM; 102630; gene.
```

```
DR   MIM; 607371; phenotype.
DR   Orphanet; 79107; Developmental malformations - deafness - dystonia.
DR   PharmGKB; PA24457; -.
DR   eggNOG; prNOG18221; -.
DR   HOVERGEN; HBG003771; -.
DR   InParanoid; P60709; -.
DR   OMA; ADSEDIQ; -.
DR   PhylomeDB; P60709; -.
DR   Reactome; REACT_17015; Metabolism of proteins.
DR   Reactome; REACT_20676; Cell junction organization.
DR   Reactome; REACT_9480; Gap junction trafficking and regulation.
DR   NextBio; 253; -.
DR   ArrayExpress; P60709; -.
DR   Bgee; P60709; -.
DR   CleanEx; HS_ACTB; -.
DR   Genevestigator; P60709; -.
DR   GermOnline; ENSG00000075624; Homo sapiens.
DR   GO; GO:0005829; C:cytosol; EXP:Reactome.
DR   GO; GO:0070688; C:MLL5-L complex; IDA:UniProtKB.
DR   GO; GO:0035267; C:NuA4 histone acetyltransferase complex; IDA:UniProtKB.
DR   GO; GO:0030529; C:ribonucleoprotein complex; IDA:UniProtKB.
DR   GO; GO:0005524; F:ATP binding; IEA:UniProtKB-KW.
DR   GO; GO:0019894; F:kinesin binding; IPI:UniProtKB.
DR   GO; GO:0050998; F:nitric-oxide synthase binding; IPI:UniProtKB.
DR   GO; GO:0005200; F:structural constituent of cytoskeleton; TAS:UniProtKB.
DR   GO; GO:0006928; P:cellular component movement; TAS:UniProtKB.
DR   InterPro; IPR004000; Actin-like.
DR   InterPro; IPR020902; Actin/actin-like_CS.
DR   InterPro; IPR004001; Actin_CS.
DR   PANTHER; PTHR11937; Actin_like; 1.
DR   Pfam; PF00022; Actin; 1.
DR   PRINTS; PR00190; ACTIN.
DR   SMART; SM00268; ACTIN; 1.
DR   PROSITE; PS00406; ACTINS_1; 1.
DR   PROSITE; PS00432; ACTINS_2; 1.
DR   PROSITE; PS01132; ACTINS_ACT_LIKE; 1.
PE   1: Evidence at protein level;
KW   3D-structure; Acetylation; ATP-binding; Complete proteome; Cytoplasm;
KW   Cytoskeleton; Deafness; Direct protein sequencing; Disease mutation;
KW   Dystonia; Methylation; Nucleotide-binding; Phosphoprotein;
KW   Polymorphism.
FT   CHAIN         1    375       Actin, cytoplasmic 1.
FT                                /FTId=PRO_0000367073.
FT   INIT_MET      1      1       Removed; alternate.
FT   CHAIN         2    375       Actin, cytoplasmic 1, N-terminally
FT                                processed.
FT                                /FTId=PRO_0000000771.
FT   MOD_RES       1      1       N-acetylmethionine; in Actin, cytoplasmic
FT                                1; alternate (By similarity).
FT   MOD_RES       2      2       N-acetylaspartate; in Actin, cytoplasmic
FT                                1, N-terminally processed.
FT   MOD_RES      53     53       Phosphotyrosine.
FT   MOD_RES      73     73       Tele-methylhistidine (By similarity).
```

```
FT   MOD_RES      91     91       Phosphotyrosine.
FT   MOD_RES     166    166       Phosphotyrosine.
FT   MOD_RES     169    169       Phosphotyrosine.
FT   MOD_RES     198    198       Phosphotyrosine.
FT   MOD_RES     218    218       Phosphotyrosine.
FT   MOD_RES     294    294       Phosphotyrosine.
FT   MOD_RES     318    318       Phosphothreonine.
FT   VARIANT     183    183       R -> W (in DYTJ; modifies cell response
FT                                to latrunculin A).
FT                                /FTId=VAR_030026.
FT   VARIANT     243    243       P -> L (in dbSNP:rs11546899).
FT                                /FTId=VAR_048185.
FT   CONFLICT     97     97       A -> P (in Ref. 6; AAH16045).
FT   CONFLICT    116    116       R -> L (in Ref. 6; AAH12854).
SQ   SEQUENCE   375 AA;  41737 MW;  6AFD05CA94E360E2 CRC64;
     MDDDIAALVV DNGSGMCKAG FAGDDAPRAV FPSIVGRPRH QGVMVGMGQK DSYVGDEAQS
     KRGILTLKYP IEHGIVTNWD DMEKIWHHTF YNELRVAPEE HPVLLTEAPL NPKANREKMT
     QIMFETFNTP AMYVAIQAVL SLYASGRTTG IVMDSGDGVT HTVPIYEGYA LPHAILRLDL
     AGRDLTDYLM KILTERGYSF TTTAEREIVR DIKEKLCYVA LDFEQEMATA ASSSSLEKSY
     ELPDGQVITI GNERFRCPEA LFQPSFLGME SCGIHETTFN SIMKCDVDIR KDLYANTVLS
     GGTTMYPGIA DRMQKEITAL APSTMKIKII APPERKYSVW IGGSILASLS TFQQMWISKQ
     EYDESGPSIV HRKCF
```
